# Supplementary material for: Three-dimensionally-printed biphasic PCL/β-TCP scaffold with spatially confined GelMA/CS hydrogel for coordinated osteochondral regeneration
Source: Regen Biomater. 2026 Jun 5;13:rbag111. doi: 10.1093/rb/rbag111 (PMC13303298; doi:10.1093/rb/rbag111)
Supplement: rbag111_Supplementary_Data [file rbag111_supplementary_data.zip › clean ZF-manuscript6.docx]

**3D-Printed Biphasic PCL/β-TCP Scaffold with Spatially Confined GelMA/CS Hydrogel for Coordinated** **Osteochondral Regeneration**

Feng Zhou^1, 2^, Xiaoyun Pan^3^, Qixiang Yin^2^, Xiao Yang^4,*^, Xiangdong Zhu^4^, Maria Grazia Raucci^5^, Luigi Ambrosio^5^, Xingdong Zhang^4^, Jingyi Mi^3,*^

^1^ Suzhou Medical College of Soochow University, Suzhou 215123, China;

^2^ Department of Emergency Surgery, Affiliated Hospital of Jiangsu University, Zhenjiang 212001, China;

^3^ Wuxi Ninth People's Hospital, Affiliated to Soochow University, Wuxi 214000, China;

^4^ National Engineering Research Center for Biomaterials, Sichuan University, Chengdu 610064, China

^5^ Institute of Polymers, Composites and Biomaterials, National Research Council, Naples 80072, Italy

*Corresponding Authors

Tel.: 86-28-85417654;

Fax: 86-28-85410246

E-mail address: xiaoyang114@scu.edu.cn (Xiao Yang) and mijingyi@suda.edu.cn (Jingyi Mi)

**Abstract**

Osteochondral defects remain difficult to repair because articular cartilage and subchondral bone differ in structure and regenerative capacity, and stable interface integration is challenging. Here, we developed a structurally continuous yet functionally stratified biphasic scaffold via dual-temperature 3D printing, consisting of a PCL/β-TCP bone-mimetic phase with larger macropores and a pure PCL cartilage-guiding phase with smaller pores. A GelMA/chondroitin sulfate (GelMA/CS) hydrogel was selectively infiltrated into the upper region by immersion and photocrosslinking, while a ~0.5 mm hydrogel-free transitional zone was preserved as a structural transition region between the chondral hydrogel compartment and the subchondral scaffold. In vitro, GelMA/CS functionalization enhanced chondrocyte adhesion, promoted sGAG secretion, and upregulated chondrogenic genes. Under osteochondral induction, the scaffolds supported osteo- and chondrogenic marker expression in human umbilical cord-derived mesenchymal stem cells. Transcriptomic profiling indicated enrichment of ECM- and mechanotransduction-related pathways, consistent with the involvement of TRPV4-associated mechanosensing and PI3K/AKT-related signaling. In a rabbit femoral osteochondral defect model, the GelMA/CS-functionalized scaffold enhanced early subchondral bone regeneration by micro-CT and promoted cartilage-like matrix deposition and chondrogenic marker staining compared with the unmodified scaffold and untreated control at 4 and 8 weeks. This hydrogel-integrated biphasic design offers a scalable strategy for coordinated osteochondral regeneration.

**1. Introduction**

Osteochondral defects, commonly caused by trauma, degenerative joint disease, or age-related degeneration, involve concurrent injury to articular cartilage and the underlying subchondral bone [1, 2]. Repair remains clinically challenging due to the stark differences in composition, vascularity, cellularity, and regenerative potential between these two tissues [3, 4]. Articular cartilage is avascular, alymphatic, and sparsely cellular, exhibiting minimal intrinsic repair capacity, whereas subchondral bone is highly vascularized and continuously remodeled. This mismatch often leads to asynchronous healing and poor osteochondral integration, ultimately accelerating joint degeneration and contributing to osteoarthritis if left unresolved [5]. Current clinical options—including microfracture, autologous chondrocyte implantation, and osteochondral autograft/allograft transplantation—may offer short-term symptomatic relief. However, long-term outcomes are frequently compromised by fibrocartilage formation, donor-site morbidity, limited graft availability, and insufficient integration at the cartilage–bone interface [6, 7]. These limitations have fueled growing interest in osteochondral tissue engineering, which aims to restore the native osteochondral unit by recapitulating its zonal architecture, biomechanical gradients, and biological functionality through biomimetic scaffold design [8, 9]. Among scaffold-based approaches, biphasic and multilayered constructs that spatially combine osteoinductive and chondroinductive compartments have shown promise for region-specific regeneration [10, 11]. However, major translational barriers remain, including weak interlayer adhesion, mismatched degradation kinetics, limited load-bearing capacity under physiological conditions, and incomplete functional integration at the interface [12]. These challenges underscore the need for advanced scaffold systems that not only mimic the hierarchical complexity of native osteochondral tissue, but also ensure structural continuity and coordinated biological responses across the cartilage–bone continuum.

Among synthetic polymers used in osteochondral tissue engineering, poly(ε-caprolactone) (PCL) is widely adopted for the osseous scaffold phase due to its thermoplastic processability, mechanical strength, and slow hydrolytic degradation [13, 14]. These properties make PCL highly compatible with extrusion-based additive manufacturing platforms, such as fused deposition modeling (FDM) and melt electrowriting (MEW), allowing precise control over pore geometry, anisotropic mechanical behavior, and patient-specific designs. However, native PCL is biologically suboptimal—its hydrophobic surface and lack of intrinsic cell-adhesive motifs can limit early cellular attachment, proliferation, and osteogenic differentiation [15]. To address these limitations, β-tricalcium phosphate (β-TCP), a bioresorbable and osteoconductive ceramic, is frequently incorporated into the PCL matrix [16, 17]. β-TCP not only enhances scaffold hydrophilicity but also provides sustained release of calcium and phosphate ions that activate osteogenic signaling pathways and promote mesenchymal stem cell (MSC) commitment, including upregulation of RUNX2, ALP, and OCN. At moderate loading (e.g., 20 wt%), polymer/β-TCP composites have demonstrated improved osteogenic marker expression alongside favorable mechanical properties (~28 MPa) [18]. However, increasing β-TCP content beyond ~30 wt% can impair filament cohesion and printing fidelity, underscoring the trade-off between enhanced bioactivity and printability. Beyond compositional tuning, hierarchical architecture across multiple length scales has been explored to better mimic the anisotropic and porous nature of native subchondral bone. Dual-resolution printing strategies—combining FDM-generated macropores (~340 μm) with MEW-deposited microfibers (~40 μm)—enable the fabrication of continuous, multiscale scaffolds that increase surface area, improve nutrient diffusion, and promote cell–material interactions [19]. While such designs support more uniform tissue infiltration and osteogenic differentiation, they also increase fabrication complexity [20]. This highlights the need for scalable scaffold strategies that strike a balance between biological functionality and manufacturing efficiency.

Reconstructing the cartilage compartment in osteochondral scaffolds remains challenging due to its distinct hydration, mechanical softness, and complex biochemical signaling. Hydrogel-based systems have thus emerged as promising candidates for mimicking the viscoelastic extracellular matrix (ECM) of native cartilage. Among them, gelatin methacryloyl (GelMA) is widely used due to its rapid and controllable photopolymerization, tunable stiffness, and retention of bioactive motifs such as RGD sequences and MMP-sensitive domains [21, 22]. These properties support chondrocyte viability and phenotype maintenance, and promote mesenchymal stem cell (MSC) chondrogenesis, as reflected by increased sulfated glycosaminoglycan (sGAG) and type II collagen expression [23, 24]. However, pristine GelMA hydrogels often suffer from insufficient mechanical robustness and rapid enzymatic degradation under physiological loading. To enhance stability and incorporate cartilage-mimetic biochemical cues, chondroitin sulfate (CS)—a sulfated glycosaminoglycan abundant in native cartilage ECM—has been incorporated into GelMA to form hybrid networks [25, 26]. CS improves hydration and crosslink density, strengthens structural integrity, and activates chondrogenic gene programs (e.g., SOX9, ACAN, COL2A1), while offering context-dependent immunomodulatory effects. These benefits are highly formulation-dependent, emphasizing the need for tailored design based on CS content, methacrylation degree, and culture conditions. From a translational perspective, top-down infiltration of GelMA-based hydrogels into 3D-printed thermoplastic lattices enables precise localization of the chondral phase without compromising the scaffold’s mechanical function. Previous studies report that such hybrid constructs enhance surface hydrophilicity, improve cell–material interactions, and can promote early ECM deposition and vascularized tissue ingrowth [27]. Furthermore, their lubricious character may recapitulate the boundary-lubricating function of superficial cartilage, potentially mitigating friction-induced tissue damage post-implantation [28].

Herein, we developed a structurally continuous yet functionally compartmentalized biphasic scaffold by integrating a mechanically robust PCL/β-TCP subchondral framework with a hydrogel-functionalized cartilage phase via spatially confined top-down infiltration. This design strategy aims to emulate native osteochondral zonation without introducing discrete calcified barrier layers, while preserving structural continuity and enabling region-specific biological cues. We systematically characterized the scaffold’s structural and physicochemical properties, and evaluated its biological performance via in vitro co-culture with human articular chondrocytes and umbilical cord–derived mesenchymal stem cells (hUCMSCs), focusing on cell adhesion, migration, and lineage-specific differentiation. RNA sequencing was conducted to explore scaffold-associated transcriptomic programs related to osteogenic and chondrogenic responses. The scaffold’s reparative potential was further assessed in a rabbit osteochondral defect model using micro-CT, histological, and immunohistochemical analyses. This study presents a scalable strategy that leverages hierarchical architecture and spatially controlled microenvironmental modulation to achieve coordinated osteochondral regeneration.

**2. Materials and methods**

**2.1 Fabrication of biphasic PCL/β-TCP scaffolds**

Biphasic osteochondral scaffolds (5 mm in diameter, 5 mm in total height) were fabricated via a sequential, dual-temperature extrusion-based 3D-printing process. The constructs consisted of a 3 mm bone-mimetic phase composed of a poly(ε-caprolactone)/β-tricalcium phosphate (PCL/β-TCP) composite and a 2 mm cartilage-guiding phase of pure PCL. To create a stepwise pore-size grading, the bone and cartilage phases were printed with distinct strand spacings (600 and 400 μm, respectively), yielding average pore sizes of ~300 μm and ~200 μm. Cylindrical scaffold models were designed in SolidWorks and sliced using Bioplotter RP software (EnvisionTEC) with a layer height of 240 μm. G-code was generated in VisualMachines, and all layers were deposited in a 0°/90° alternating orthogonal grid pattern.

**Bone-phase ink preparation and low-temperature extrusion**

PCL pellets (Mw ≈ 80 kDa; Sigma-Aldrich) were vacuum-dried at 25 °C for 30 min prior to use. Dried PCL (0.6 g) was dissolved in dichloromethane (DCM, 6 mL) under magnetic stirring at ~21 °C for 1 h. After complete dissolution, absolute ethanol (2 mL) was added to adjust viscosity and improve miscibility (DCM/EtOH = 3:1, v/v), followed by stirring for an additional 30 min. Nano-β-TCP powder was then gradually incorporated to achieve a ceramic loading of ~80 wt% (PCL:β-TCP = 1:4, w/w). The composite ink was loaded into a 20 mL syringe and sequentially passed through needles of decreasing inner diameters (800, 640, and 410 μm) to remove agglomerates and facilitate partial solvent evaporation, yielding a viscous, paste-like ink suitable for low-temperature extrusion. The bone phase was printed using a low-temperature extrusion printer (CLRF-2000-II, Tsinghua University) at 20 °C through a 300 μm nozzle, with an extrusion pressure of 1.8–2.2 bar and a printing speed of 10–15 mm s⁻¹. A 0°/90° grid infill with a strand spacing of 600 μm was used, resulting in pores of ~300 μm.

**Cartilage-phase melt extrusion printing**

Immediately after fabrication of the bone phase, the cartilage phase was printed directly on top by melt extrusion of pure PCL. Vacuum-dried PCL pellets (2 g) were loaded into a high-temperature print head preheated to 80 °C and thermally equilibrated for 30 min. Extrusion was performed through a 300 μm nozzle at 3.8 bar with a printing speed of 0.7 mm s⁻¹. Printing proceeded without intermediate cooling to promote interfacial fusion between the two phases. The cartilage layer was printed with a strand spacing of 400 μm, generating pores of ~200 μm.

**Post-processing**

Completed biphasic scaffolds were dried in an oven at 40 °C for 4 h to remove residual DCM and ethanol from the bone-phase region.

**2.2 Preparation of GelMA/CS-coated scaffolds**

Gelatin methacryloyl (GelMA; EFL, Engineering For Life, China) was dissolved in phosphate-buffered saline (PBS) containing 0.05% (w/v) lithium phenyl-2,4,6-trimethylbenzoylphosphinate (LAP) at 40 °C under gentle stirring until a clear solution was obtained. Chondroitin sulfate (CS; Sigma-Aldrich) was then added to achieve final concentrations of 6% (w/v) GelMA and 3% (w/v) CS, yielding a homogeneous prepolymer solution after thorough mixing. To functionalize the cartilage-guiding phase, pre-fabricated biphasic scaffolds were pre-warmed to 37 °C and vertically immersed from the cartilage side into the GelMA/CS prepolymer for 3 min. Capillary-driven infiltration enabled the precursor to penetrate the upper PCL lattice to a controlled depth of ~1.5 mm, which was regulated by the immersion level and contact time. A ~0.5 mm uninfiltrated PCL transitional zone was deliberately retained between the hydrogel-filled cartilage region and the underlying bone-mimetic phase, forming a structural transition region between the chondral hydrogel compartment and the subchondral scaffold. Photocrosslinking was carried out by UV irradiation (365 nm, 6 mW cm⁻²) for 30 s, forming an in situ crosslinked GelMA/CS hydrogel network anchored within the macropores of the PCL scaffold. The constructs were thoroughly rinsed with PBS to remove uncrosslinked residues and stored in PBS at 4 °C until further use. All procedures were performed under sterile conditions. Separate hydrogel samples prepared under the same formulation conditions were used for rheological characterization, and integrated biphasic constructs were used for cyclic mechanical testing.

**2.3 Structural and physicochemical characterization**

The surface morphology, pore architecture, and interfacial integration between the cartilage-guiding and bone-mimetic regions were characterized using field-emission scanning electron microscopy (FE-SEM; SUPRA 55, Zeiss, Germany) after gold sputter-coating. Both top-view and cross-sectional images were acquired to assess filament arrangement, pore size distribution, and the continuity of interlayer fusion. Quantitative measurements of filament widths and pore diameters were performed using ImageJ (NIH, USA). Elemental composition and spatial distribution were analyzed via energy-dispersive X-ray spectroscopy (EDS; X-Max 20, Oxford Instruments, UK) integrated with the SEM. Elemental mapping of calcium (Ca), phosphorus (P), and carbon (C) was used to verify homogeneous β-TCP dispersion within the PCL matrix. Chemical composition was evaluated by attenuated total reflectance Fourier-transform infrared spectroscopy (ATR-FTIR; Nicolet iS50, Thermo Fisher Scientific, USA) and X-ray diffraction (XRD, Bruker D8 Advance, Germany). Hydrogel infiltration was examined by SEM imaging of lyophilized, cryo-fractured GelMA/CS-coated scaffolds. Mechanical properties of the fully integrated biphasic scaffolds (PCL/β-TCP base with GelMA/CS hydrogel coating) were first evaluated under unconfined compression using a universal testing machine (Instron 5943, USA). Cylindrical samples (5 mm diameter × 5 mm height) were compressed at a constant rate of 1 mm min⁻¹, and the compressive modulus was determined from the linear region (0–10% strain) of the stress–strain curve. To further assess construct-level behavior under repeated loading, cyclic compression testing was performed on representative scaffold-only and hydrogel-integrated constructs using a mechanical testing system. Samples were subjected to repeated compressive loading, and the load–deformation hysteresis behavior was recorded. The first loading cycle was treated as a conditioning cycle, and the subsequent cycles were used to evaluate cyclic stability and viscoelastic energy dissipation of the constructs. Rheological properties of GelMA and GelMA/CS hydrogels were measured using a rotational rheometer (MCR 92, Anton Paar, Austria) equipped with RheoCompass software at 25 °C. Flow sweep tests were performed over a shear rate range of 0.1–100 s⁻¹ to determine the relationship between viscosity, shear stress, and shear rate. Oscillatory strain sweep tests were subsequently carried out to determine the storage modulus (G′), loss modulus (G″), and loss factor (tan δ) as a function of strain. These measurements were used to evaluate shear-thinning behavior and strain-dependent viscoelasticity of the hydrogel formulations.

**2.4 In vitro chondrocyte culture on GelMA and GelMA/CS hydrogels**

Primary human articular chondrocytes (hACs; Procell Life Science & Technology Co., Ltd., Wuhan, China; Cat. No. CP-H107) were expanded and maintained according to the supplier’s protocol. Cells were cultured in the manufacturer-provided chondrocyte-specific growth medium at 37 °C in a humidified atmosphere containing 5% CO₂. Low-passage cells (passages 2–3) were used for all experiments to preserve the chondrogenic phenotype. To evaluate chondrocyte responses to the hydrogel surfaces, a direct seeding model was employed. Sterilized GelMA and GelMA/CS hydrogel samples were placed in 24-well plates, and hACs were seeded onto the hydrogel surfaces at a density of 1 × 10⁵ cells per well. For the control group, hACs were cultured on tissue-culture plastic under the same seeding density and culture conditions. After 4 h of initial attachment in a minimal volume of medium, additional complete growth medium was added. Cultures were maintained with medium changes every 2–3 days. Cell proliferation was assessed using the Cell Counting Kit-8 (CCK-8) assay at days 1, 4, and 7. Samples were incubated with 10% (v/v) CCK-8 working solution for 2 h at 37 °C, and absorbance was measured at 450 nm using a microplate reader. Cytoskeletal organization was assessed at 48 h after seeding. Samples were fixed with 4% paraformaldehyde (PFA) in PBS for 15 min at room temperature, permeabilized with 0.1% Triton X-100 for 10 min, and blocked with 1% bovine serum albumin (BSA) in PBS for 60 min. F-actin was stained with FITC-conjugated phalloidin (1:200 dilution; Thermo Fisher Scientific) for 60 min at room temperature in the dark, and nuclei were counterstained with DAPI (1 μg mL⁻¹) for 5 min. Fluorescence images were acquired using a confocal laser scanning microscope (LSM 710, Zeiss, Germany). Chondrogenic matrix production was quantified by measuring secreted sulfated glycosaminoglycans (sGAG) in culture supernatants. Supernatants were collected on days 14 and 21, centrifuged to remove debris, and analyzed using a human sGAG ELISA kit (Shanghai Enzyme-linked Biotechnology Co., Ltd., China) according to the manufacturer’s instructions. To normalize for variations in cell number, sGAG levels were normalized to total DNA content, which was quantified from corresponding cell lysates using the PicoGreen dsDNA Quantitation Kit (Thermo Fisher Scientific, USA). All experiments were performed in triplicate with at least three independent biological replicates.

**2.5 In vitro chondrocyte migration assay**

Chondrocyte migration was evaluated using a scratch wound-healing assay to compare the pro-migratory effects of GelMA and GelMA/CS hydrogel extracts. Primary human articular chondrocytes (hACs; passages 2–3) were seeded into 6-well plates and cultured to confluence in complete chondrocyte growth medium. A linear scratch was introduced across the cell monolayer using a sterile 200 μL pipette tip. Detached cells and debris were removed by washing gently with PBS (×3). Wounded monolayers were then incubated in low-serum medium (DMEM supplemented with 1% fetal bovine serum, FBS) containing either GelMA extract, GelMA/CS extract, or control medium (low-serum DMEM only). Hydrogel extracts were prepared by incubating sterilized GelMA or GelMA/CS hydrogels in serum-free DMEM at 37 °C for 24 h under gentle agitation. A surface area-to-volume ratio of 1 cm² per 1 mL of medium was used. The supernatants were collected, centrifuged (1000 × g, 5 min), and filtered through a 0.22 μm syringe filter to remove residual particulates. The resulting extracts were supplemented directly into low-serum medium at 100% (v/v) concentration. Phase-contrast images were acquired at 0, 6, 12, and 24 h using an inverted microscope at predefined positions marked with reference lines on the plate underside. Wound closure was quantified using ImageJ. The percentage of wound closure was calculated using the equation: Wound closure (%) = ((A₀ − A_t_) / A₀) × 100, where A₀ is the wound area at time 0, and A_t_ is the area at time *t*. Quantification was performed on at least three independent fields per well, and all experiments were conducted in triplicate with six biological replicates.

**2.6 In vitro evaluation of hUCMSC responses to scaffold**

Human umbilical cord-derived mesenchymal stem cells (hUCMSCs; Yimo Cell Bank, Cat. No. IMP-H122) at passages 3–5 were used to investigate the effects of scaffold composition and hydrogel functionalization on cell behavior under osteochondral induction conditions. Cells were expanded according to the manufacturer’s protocol in complete growth medium at 37 °C in a humidified atmosphere containing 5% CO₂. Before scaffold-based experiments, hydrogel formulations with different GelMA and CS concentrations were preliminarily screened using hydrogel-conditioned medium. hUCMSCs were cultured with conditioned medium prepared from GelMA/CS hydrogels containing 5%, 6%, or 7% GelMA (w/v) combined with different CS concentrations, and cell proliferation was assessed by CCK-8 at days 1, 3, and 5. Based on the initial screening, formulations containing 6% GelMA were further evaluated by qRT-PCR for chondrogenic gene expression (SOX9, COL2A1, and ACAN), and 6% GelMA/3% CS was selected for subsequent scaffold functionalization. Four experimental groups were established: (i) control (cells only, no scaffold), (ii) uncoated biphasic scaffold (PCL/β-TCP), (iii) GelMA-integrated biphasic scaffold (PCL/β-TCP@GelMA), and (iv) GelMA/CS-integrated biphasic scaffold (PCL/β-TCP@GelMA/CS). Scaffolds were fabricated as described in Section 2.1, and hydrogel integration into the cartilage phase was achieved by controlled top-side immersion followed by UV photocrosslinking (365 nm, 6 mW cm⁻², 30 s). Scaffolds were washed five times with sterile PBS and preconditioned overnight in complete growth medium at 37 °C. hUCMSCs were seeded onto the cartilage-guiding (upper) surface at 1 × 10⁶ cells per scaffold in a 100 μL droplet and allowed to attach for 4 h under static conditions (37 °C, 5% CO₂). Constructs were then transferred to 24-well plates and submerged in osteochondral induction medium, which consisted of high-glucose DMEM supplemented with 10% FBS, 1% penicillin–streptomycin, 50 μg mL⁻¹ ascorbic acid 2-phosphate, 10 mM β-glycerophosphate, 100 nM dexamethasone, and 10 ng mL⁻¹ recombinant human TGF-β1. Medium was refreshed every 2–3 days, and cultures were maintained for up to 7 days. Cell proliferation was assessed using the Cell Counting Kit-8 (CCK-8; Dojindo) assay at days 1, 3, and 5. Samples were incubated with 10% (v/v) CCK-8 working solution for 2 h at 37 °C, and 100 μL aliquots were transferred to a 96-well plate for absorbance measurement at 450 nm using a microplate reader (Bio-Rad). Data were normalized to day 1 values and presented as relative proliferation. At day 7, sGAG levels in culture supernatants were quantified using a commercial ELISA kit following the manufacturer’s instructions. Absorbance was read at 450 nm, and sGAG content was normalized to total DNA quantified from corresponding samples using the PicoGreen dsDNA Quantitation Kit (Thermo Fisher Scientific, USA). All assays were performed in triplicate with at least six independent biological replicates (n ≥ 6 per group).

**2.7 Transcriptomic sequencing analysis**

To explore scaffold-associated transcriptomic changes in hUCMSCs, RNA sequencing (RNA-seq) was performed after 14 days of osteochondral induction. Two experimental groups were analyzed: (i) control (no scaffold) and (ii) PCL/β-TCP@GelMA/CS (biphasic scaffold with GelMA/CS hydrogel integrated in the cartilage phase). hUCMSCs were cultured under osteochondral induction conditions for 14 days prior to RNA isolation. RNA-seq was performed using two biological replicates per group (n = 2). Total RNA was extracted using the RNeasy Mini Kit (Qiagen, Germany) according to the manufacturer’s protocol. Cells were lysed in RLT buffer, and RNA integrity was assessed by agarose gel electrophoresis. RNA concentration and purity were determined using a NanoDrop ND-1000 spectrophotometer (Thermo Fisher Scientific, USA). RNA-seq library preparation, sequencing, and primary processing were conducted by an external service provider (Wuhan Benagen Technology Co., Ltd., Wuhan, China). Libraries were prepared using the NEBNext® Ultra™ RNA Library Prep Kit and sequenced on an Illumina NovaSeq 6000 platform to generate paired-end reads (PE150). The libraries generated approximately 19.92–20.01 million clean paired-end reads per sample. Raw reads were subjected to quality control using FastQC and fastp to remove adaptor sequences and low-quality reads. Clean reads were aligned to the human reference genome (GRCh38) using HISAT2. Gene-level read counts were generated with featureCounts, and differential expression analysis was performed using DESeq2. Genes meeting the criteria of |log₂ fold change| ≥ 1 and adjusted p-value (padj) < 0.05 were defined as differentially expressed genes (DEGs). Functional annotation of DEGs was performed using Gene Ontology (GO) and Kyoto Encyclopedia of Genes and Genomes (KEGG) enrichment analyses to identify significantly altered biological processes and pathways. In addition, Gene Set Enrichment Analysis (GSEA) was conducted to evaluate coordinated changes across predefined gene sets and to interrogate broader transcriptomic programs associated with the response to the GelMA/CS-functionalized scaffold.

**2.8 Quantitative real-time PCR analysis**

Quantitative real-time PCR (qRT-PCR) was performed to assess gene expression associated with chondrogenic and osteogenic differentiation and to validate RNA-seq findings. Three experimental settings were analyzed: (i) hACs cultured on GelMA or GelMA/CS hydrogels for 14 days under chondrocyte-specific culture conditions; (ii) hUCMSCs cultured for 7 days under osteochondral induction on different substrates, including tissue-culture plastic (control), biphasic PCL/β-TCP scaffolds, PCL/β-TCP@GelMA scaffolds, and PCL/β-TCP@GelMA/CS scaffolds; and (iii) hUCMSCs cultured under the control condition (no scaffold) or on PCL/β-TCP@GelMA/CS scaffolds for RNA-seq validation. Total RNA was extracted using the RNeasy Mini Kit (Qiagen, Germany) according to the manufacturer’s instructions, and RNA concentration and purity were determined using a NanoDrop ND-1000 spectrophotometer (Thermo Fisher Scientific, USA). For each sample, 500 ng of total RNA was reverse-transcribed into cDNA using the iScript™ cDNA Synthesis Kit (Bio-Rad, USA). qRT-PCR was carried out on a QuantStudio 5 Real-Time PCR System (Thermo Fisher Scientific, USA) using SYBR Green Master Mix (Bio-Rad, USA) in 20 μL reaction volumes. The cycling conditions were: 95 °C for 30 s, followed by 40 cycles of 95 °C for 10 s and 60 °C for 60 s. A melt-curve analysis (65–95 °C, 0.5 °C s⁻¹) was performed to confirm amplification specificity. For hACs, target genes included SOX9, COL2A1, ACAN, COMP, COL1A1, and COL10A1. For hUCMSCs, osteogenic markers (RUNX2, BMP2, COL1A1, OCN, and OPN) and chondrogenic markers (SOX9, COL2A1, and ACAN) were analyzed. For RNA-seq validation, GLI1, TRPV4, PRG4, HIF3A, FGF18, and FGFR3 were quantified. GAPDH served as the internal reference gene, and relative expression levels were calculated using the 2^−ΔΔCt method. Primer sequences are provided in Supplementary Table S1.

**2.9 Western blot analysis**

Western blotting was performed to validate selected RNA-seq-associated targets and to assess TRPV4- and PI3K/AKT-related signaling changes. hUCMSCs were divided into four experimental groups: (i) control (no scaffold), (ii) PCL/β-TCP@GelMA/CS scaffold, (iii) PCL/β-TCP@GelMA/CS scaffold with TRPV4 inhibitor (RN-1734, 10 μM; MedChemExpress, #HY-100289), and (iv) PCL/β-TCP@GelMA/CS scaffold with PI3K inhibitor (LY294002, 10 μM; Sigma-Aldrich, #L9908). Cells were cultured for 48 h under osteochondral induction conditions as described in Section 2.6. Total protein was extracted using RIPA lysis buffer (Beyotime, China) supplemented with protease and phosphatase inhibitor cocktails (Roche, Switzerland), and protein concentrations were determined using a BCA Protein Assay Kit (Bio-Rad, USA). Equal amounts of protein (30 μg per lane) were separated by 12% SDS–PAGE and transferred onto PVDF membranes (Millipore, USA) by wet transfer (350 mA, 2 h). Membranes were blocked with 5% non-fat dry milk in TBST (0.1% Tween-20 in TBS) for 2 h at room temperature and incubated overnight at 4 °C with primary antibodies diluted 1:1000 in 5% BSA/TBST. For validation of scaffold-responsive targets and osteochondral differentiation-associated proteins, membranes were probed with antibodies against TRPV4 (CST, #65893), SOX9 (CST, #82630), ACAN (CST, #28971), COL2A1 (CST, #43306), RUNX2 (CST, #12556), OCN (CST, #59757), PRG4 (HUABIO, #ER1912-53), and GAPDH (Bioworld, #AP0063). For pathway-related analysis under inhibitor treatment conditions, additional membranes were probed with antibodies against AKT (CST, #9272), phospho-AKT (Ser473) (CST, #4060), PI3K p85 (CST, #4292), and phospho-PI3K p85 (Tyr458)/p55 (Tyr199) (CST, #4228). After washing, membranes were incubated with HRP-conjugated secondary antibodies (CST, 1:3000) for 1 h at room temperature. Protein bands were visualized using enhanced chemiluminescence (ECL; Thermo Fisher Scientific, USA) and imaged using the ChemiDoc MP imaging system (Bio-Rad, USA). Densitometric analysis, where applicable, was performed using ImageJ, and protein expression levels were normalized to GAPDH.

**2.10 In vivo osteochondral repair in a rabbit model**

All animal experiments were approved by the Institutional Animal Care and Use Committee of Jiangsu University (Approval No. UJS-IAUC-2023122105) and performed in accordance with national and institutional guidelines for the care and use of laboratory animals. Eighteen adult male New Zealand white rabbits (2.5–3.0 kg) were used to evaluate osteochondral repair. Three treatment conditions were included: (i) control (untreated defect), (ii) uncoated biphasic scaffold (PCL/β-TCP), and (iii) hydrogel-functionalized biphasic scaffold (PCL/β-TCP@GelMA/CS). At each time point, treatments were assigned in a randomized bilateral paired manner such that each rabbit received two different treatments, one per knee. Under general anesthesia with isoflurane, both hind limbs were shaved and disinfected. A medial parapatellar approach was used to expose the femoral trochlear groove, followed by lateral patellar dislocation. Bilateral full-thickness cylindrical osteochondral defects (5 mm in diameter × 5 mm in depth) were created in the trochlear groove using a motorized trephine under continuous saline irrigation to minimize thermal damage. At each time point, 9 rabbits were used, and each treatment group comprised 6 knees. The joint capsule was closed with absorbable sutures, and the skin was closed with nonabsorbable sutures. Postoperatively, rabbits received intramuscular penicillin (800,000 U day⁻¹) for 3 days and were housed individually with ad libitum access to food and water. At 4 and 8 weeks post-implantation, animals were anesthetized with isoflurane and euthanized by CO₂ inhalation. Distal femoral specimens were harvested, fixed in 4% paraformaldehyde for 48 h, and processed for gross observation, histological staining, and semiquantitative evaluation according to the International Cartilage Repair Society (ICRS) macroscopic and histological guidelines.

**2.11 Micro-computed tomography analysis**

To quantitatively assess subchondral bone regeneration, distal femoral specimens from the blank control, PCL/β-TCP, and PCL/β-TCP@GelMA/CS groups harvested at 4 and 8 weeks post-implantation were scanned using a high-resolution micro-computed tomography system (micro-CT 100; SCANCO Medical AG, Brüttisellen, Switzerland). Specimens were fixed in 4% paraformaldehyde and mounted vertically in custom holders to maintain consistent orientation during scanning. Scans were acquired with the following parameters: isotropic voxel size, 18 μm; X-ray tube voltage, 70 kV; tube current, 120 μA; integration time, 300 ms; and a 0.5 mm aluminum filter. Raw projection data were reconstructed into cross-sectional images and 3D volumetric datasets using the manufacturer’s software. A cylindrical volume of interest (VOI; 5.0 mm in diameter × 5.0 mm in height) was defined within the central defect region using consistent anatomical landmarks to minimize edge effects and artifacts. Mineralized tissue was segmented using global thresholding; the threshold was determined by histogram-based calibration and then uniformly applied across all samples and time points to enable direct comparisons. Bone microarchitectural parameters were quantified using Scanco evaluation software, including bone volume fraction (BV/TV, %), bone mineral density (BMD, mg HA cm⁻³), trabecular thickness (Tb.Th, μm), trabecular number (Tb.N, mm⁻¹), trabecular separation (Tb.Sp, μm), and bone volume (BV, mm³). Three-dimensional reconstructions of the defect region were generated for qualitative visualization of subchondral bone formation and bone–scaffold integration. Macroscopic score was independently scored by two blinded observers according to the International Cartilage Repair Society (ICRS) guidelines.

**2.12 Histological and immunohistochemical evaluation**

At 4 and 8 weeks post-implantation, distal femoral specimens from all groups (control, PCL/β-TCP, and PCL/β-TCP@GelMA/CS) were harvested for macroscopic observation and histological analysis. Samples were fixed in 4% paraformaldehyde (pH 7.4) for 48 h at room temperature and decalcified in 10% EDTA (pH 7.2) for 15–20 weeks, with the decalcification solution refreshed every 3 days. After thorough PBS rinsing, specimens were dehydrated through a graded ethanol series, cleared in xylene, embedded in paraffin, and sagittally sectioned through the center of the defect at a thickness of 5 μm using a rotary microtome (Leica RM2235, Germany). Paraffin sections were stained with hematoxylin and eosin (H&E) to assess overall tissue morphology and defect filling, Masson’s trichrome to evaluate collagen deposition and fibrous tissue organization, Safranin O/Fast Green for sGAG-rich cartilage matrix, and Toluidine Blue to assess cartilage metachromasia. Adjacent serial sections were used for immunohistochemical (IHC) staining to detect osteochondral repair–related proteins, including SOX9, aggrecan (ACAN), and type II collagen (COL2). For IHC, sections were deparaffinized in xylene, rehydrated through descending ethanol gradients, and subjected to heat-induced antigen retrieval using 10 mM sodium citrate buffer (pH 6.0). Endogenous peroxidase activity was blocked using 3% H₂O₂ for 10 min, followed by 30 min blocking in 5% bovine serum albumin (BSA) at room temperature. Primary antibodies (Novus Biologicals, USA; 1:300 dilution) were applied overnight at 4 °C. After PBS washing, sections were incubated with horseradish peroxidase (HRP)-conjugated secondary antibodies (Servicebio, China; 1:500) for 50 min at room temperature. Immunoreactivity was developed using a DAB chromogenic substrate kit (Boster, China), and nuclei were counterstained with hematoxylin. All stained sections were imaged or digitally scanned (CaseViewer, 3DHISTECH) under identical acquisition settings. Semi-quantitative analysis of IHC staining was performed by measuring the integrated optical density (IOD) of DAB-positive regions within predefined regions of interest (ROIs) covering both central and peripheral zones of the defect site using ImageJ (NIH, USA). Mean IOD values were used for statistical comparison between groups and time points. For preliminary in vivo biosafety evaluation, major organs including the heart, liver, spleen, lung, and kidney were harvested at the experimental endpoint, fixed in 4% paraformaldehyde, paraffin-embedded, sectioned, and stained with H&E according to standard histological procedures.

**2.13 Statistical analysis**

All quantitative data are presented as mean ± standard deviation (SD) from at least three independent biological replicates (n ≥ 3), unless otherwise stated. Statistical analyses were performed using GraphPad Prism 9.0 (GraphPad Software, San Diego, CA, USA) and SPSS 26.0 (IBM Corp., Armonk, NY, USA). Data normality was assessed using the Shapiro–Wilk test, and homogeneity of variances was evaluated using Levene’s test. For in vitro experiments, comparisons between two groups were performed using unpaired two-tailed Student’s t-tests, and comparisons among three or more groups were performed using one-way analysis of variance (ANOVA) followed by Tukey’s post hoc test when normality and homoscedasticity assumptions were met. When these assumptions were violated, data were log-transformed; if they remained unmet, non-parametric analysis using the Kruskal–Wallis test followed by Dunn’s multiple-comparisons test was conducted. For in vivo experiments, each rabbit received two different treatments, one in each knee, according to a randomized bilateral paired allocation scheme. Therefore, in vivo data at each time point were reanalyzed using a linear mixed-effects model, with treatment as a fixed effect and rabbit identity as a blocking/random factor to account for within-animal pairing. Technical replicates were averaged within each biological replicate prior to statistical testing. A two-sided p-value < 0.05 was considered statistically significant, with significance levels indicated as *p < 0.05, **p < 0.01, and ***p < 0.001.

**3. Results**

**3.1 Characterization of the biphasic scaffold**

The structural fidelity, phase-specific architecture, and compositional distribution of the 3D-printed biphasic osteochondral scaffold were characterized by gross observation, micro-computed tomography (micro-CT), scanning electron microscopy (SEM), and complementary physicochemical analyses. Macroscopically, the scaffold exhibited a distinct bilayer structure with clear visual demarcation: the upper cartilage-guiding PCL layer appeared whitish and semi-translucent, whereas the lower bone-mimetic PCL/β-TCP layer was opaque/milky white, consistent with β-TCP incorporation (Figure 1A). As designed, the two phases displayed different pore architectures, with smaller pores in the upper layer and larger pores in the lower layer. SEM further revealed phase-specific surface microtopography. The PCL layer showed smooth strut surfaces with a relatively uniform texture (pore diameter: 321.5 ± 42.6 μm; strand width: 285.6 ± 30.0 μm). In contrast, the PCL/β-TCP layer exhibited a rougher, granular strut surface with particulate features attributable to embedded/exposed β-TCP (pore diameter: 420.5 ± 32.8 μm; strand width: 323.6 ± 22.6 μm). Notably, due to the limited X-ray contrast of the radiolucent PCL phase under the current scanning and segmentation settings, micro-CT primarily visualized the β-TCP–containing subchondral layer. The mineralized layer exhibited a regular 0°/90° orthogonal lattice with a well-interconnected through-pore network. Energy-dispersive X-ray spectroscopy (EDS) mapping revealed a sharp compositional contrast between the two layers (Figure 1B). The upper PCL layer was dominated by carbon (C) signals with negligible calcium (Ca) and phosphorus (P), confirming a ceramic-free cartilage-guiding phase. In contrast, the lower PCL/β-TCP layer showed homogeneous Ca and P signals distributed throughout the struts, demonstrating uniform β-TCP dispersion within the polymer matrix. X-ray diffraction (XRD) patterns verified the crystalline integrity of each phase (Figure 1C). The PCL layer displayed characteristic semi-crystalline PCL peaks at 2θ ≈ 21.3° and 23.7°, corresponding to the (110) and (200) planes. The composite layer exhibited these PCL peaks along with additional reflections at 2θ ≈ 25.9°, 31.0°, 34.2°, and 39.5°, consistent with β-TCP (JCPDS No. 09-0169). No peak shifts or extra peaks were observed, indicating that β-TCP retained its crystallographic structure during processing and was incorporated without forming secondary crystalline phases. ATR-FTIR further confirmed the coexistence of polymer and phosphate components. Both layers showed prominent PCL bands, including C=O stretching at ~1722 cm⁻¹, ester/C–O–C stretching in the 1300–1100 cm⁻¹ region, and aliphatic C–H vibrations. The composite layer additionally displayed phosphate-related absorptions, including PO₄³⁻ ν₃ stretching (~1000–1050 cm⁻¹) and PO₄³⁻ ν₄ bending (~560–600 cm⁻¹). Compared with pure PCL, modest changes in band intensity in the carbonyl/ester region were observed in the composite, consistent with physical incorporation and interfacial interactions, without new bands indicative of chemical transformation. These results demonstrate successful fabrication of a structurally stable biphasic scaffold with distinct pore architectures, robust interfacial integrity, and uniform β-TCP incorporation in the bone-mimetic phase.


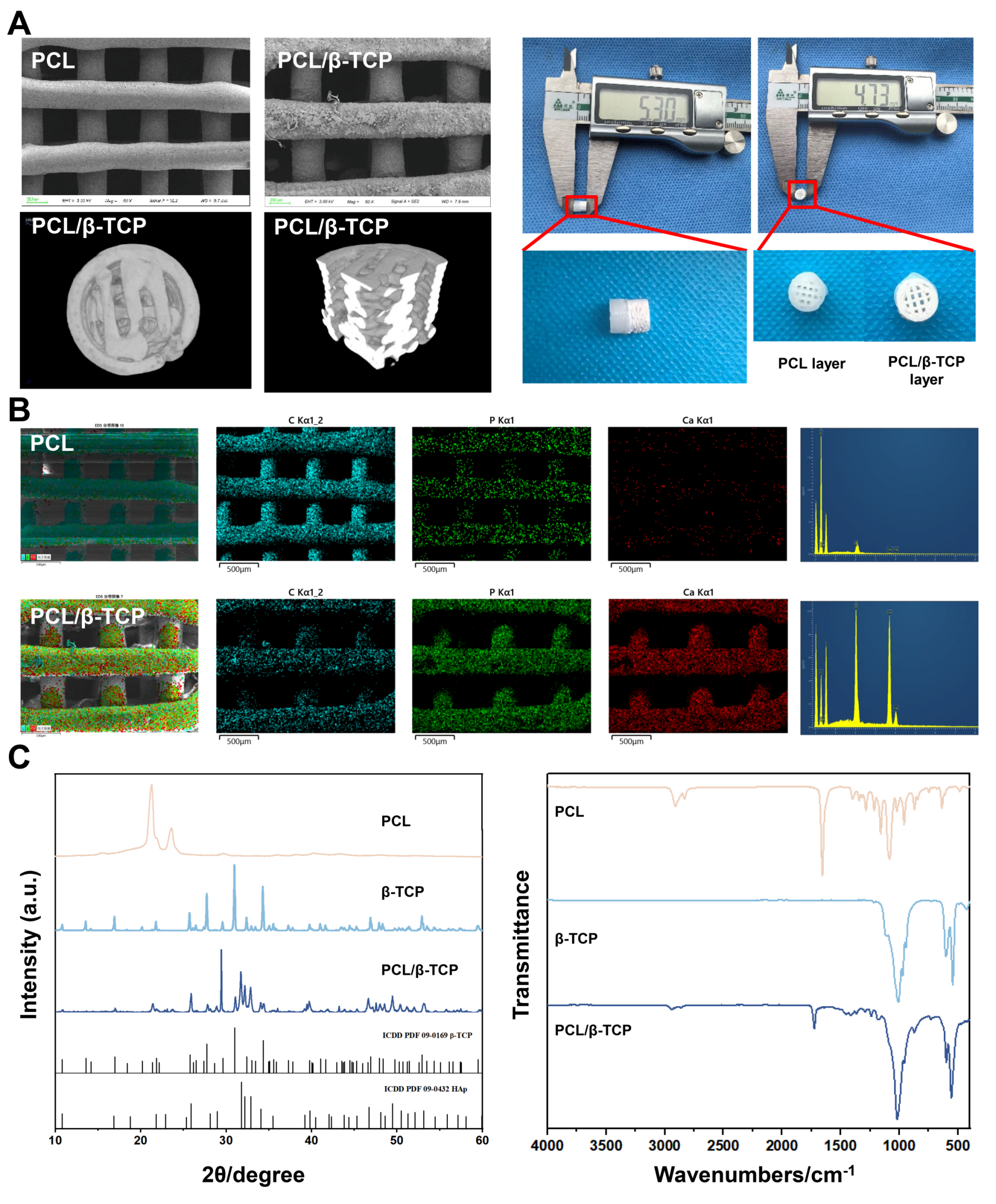


**Figure 1.** Structural and compositional characterization of the 3D-printed biphasic osteochondral scaffold. (A) Representative photographs, micro-CT 3D reconstruction (highlighting radiopaque β-TCP in the lower phase), and SEM images showing a well-defined stratified architecture with a semi-translucent upper PCL cartilage-guiding phase and an opaque lower PCL/β-TCP subchondral bone phase. (B) EDS elemental mapping of scaffold cross-sections reveals compositional segregation: dominant carbon (C) signals with negligible calcium (Ca) and phosphorus (P) in the upper PCL phase, and uniform distribution of C, Ca, and P in the lower phase, confirming homogeneous β-TCP incorporation. (C) XRD patterns of individual phases confirm characteristic diffraction peaks of PCL and β-TCP. ATR-FTIR spectra identify vibrational bands associated with PCL and phosphate groups from β-TCP, supporting phase-specific chemical composition.


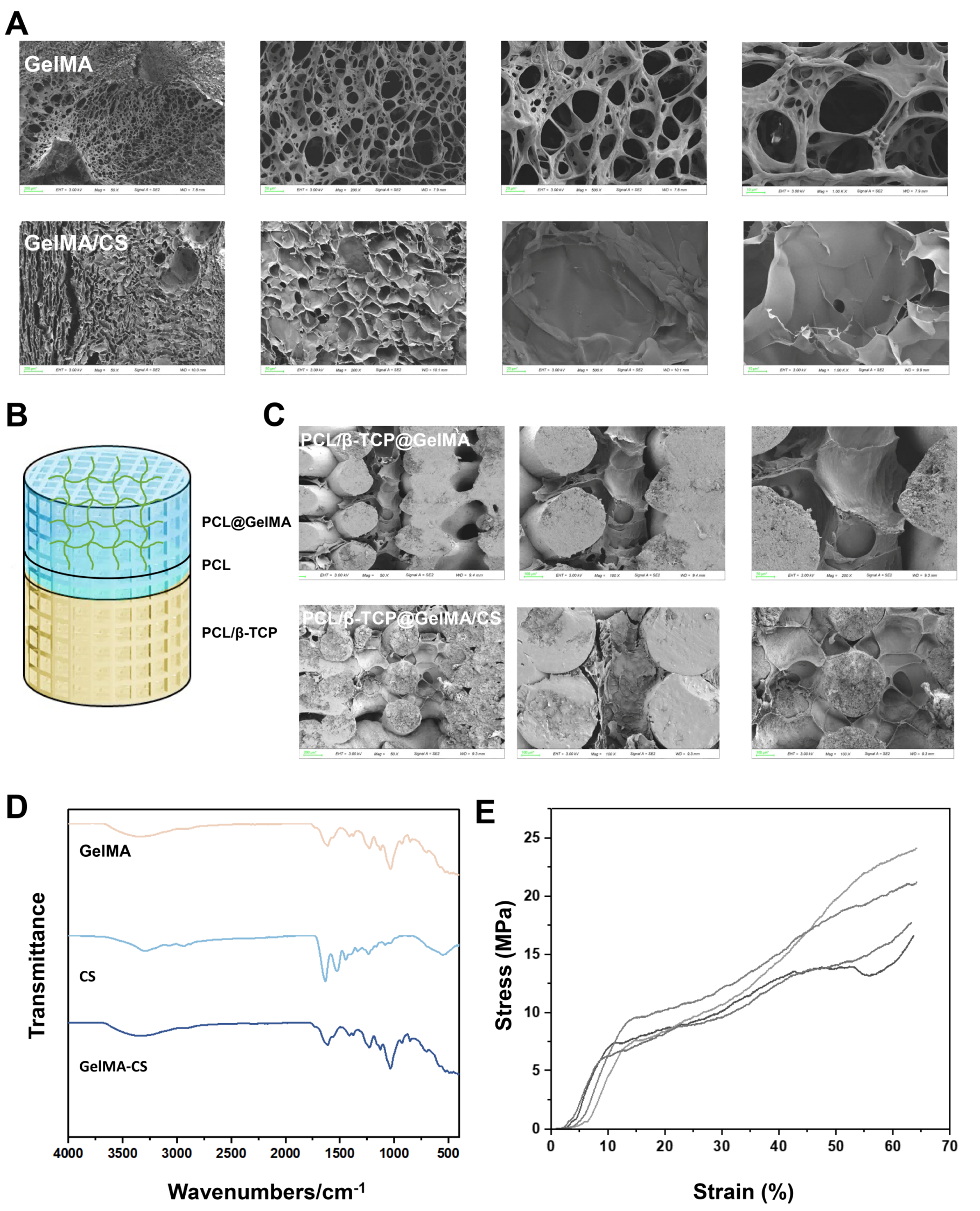


**Figure 2.** Hydrogel integration and physicochemical characterization of the biphasic scaffold. (A) SEM images of standalone GelMA and GelMA/CS hydrogels showing microporous, interconnected networks. (B) Top-view SEM of the cartilage-guiding PCL layer after spatially confined, top-down hydrogel infiltration and photocrosslinking, demonstrating uniform hydrogel retention in square macropores and close contact with PCL struts. (C) Cross-sectional SEM images reveal regionally confined hydrogel distribution within the chondral compartment and preservation of an ~0.5 mm hydrogel-free transitional zone. Compared with GelMA, GelMA/CS appears to exhibit greater matrix continuity and fewer visible interfacial voids. (D) FTIR spectra of GelMA, CS, and GelMA/CS hydrogels display characteristic amide and sulfate bands without new peak formation, indicating physical blending. (E) Representative compressive stress–strain curves of the fully integrated GelMA/CS-functionalized biphasic constructs.

**3.2 Physicochemical characterization of hydrogel-modified scaffolds**

SEM was used to examine the microstructure of the hydrogels and their integration within the biphasic scaffold. Lyophilized GelMA and GelMA/CS hydrogels exhibited an interconnected porous morphology with a honeycomb-/sponge-like architecture (Figure 2A). After controlled top-down infiltration into the upper PCL cartilage-guiding phase followed by in situ photocrosslinking, both hydrogels were uniformly retained within the square macropores and closely conformed to the internal pore walls (Figure 2B). Notably, an ~0.5 mm uninfiltrated PCL transitional zone was consistently preserved between the hydrogel-filled region and the underlying bone-mimetic phase, forming a defined structural transition region between the chondral hydrogel compartment and the subchondral scaffold. Cross-sectional SEM further confirmed deep and uniform hydrogel penetration into the chondral compartment to a controlled depth of ~1.5 mm, forming a continuous interpenetrating network within the macroporous PCL lattice (Figure 2C). The hydrogel matrices adhered closely to the PCL struts, indicating effective pore-wall conformity after infiltration and curing. Compared with GelMA alone, GelMA/CS appeared to exhibit improved matrix continuity and fewer visible interfacial voids at the hydrogel–strut boundary, consistent with more homogeneous filling within the porous framework. ATR-FTIR spectra verified the retention of characteristic functional groups from both GelMA and CS in the composite hydrogel (Figure 2D). GelMA showed a broad O–H/N–H stretching band (~3291 cm⁻¹) and a prominent amide I band (~1633 cm⁻¹), whereas CS exhibited representative absorptions at ~1034 cm⁻¹ (C–O–C stretching) and ~1227 cm⁻¹ (S=O stretching of sulfate groups), along with a band near the amide I region (~1643 cm⁻¹). In the GelMA/CS hydrogel, these signatures were simultaneously observed without the appearance of new bands, supporting physical blending while preserving the chemical identities of both components. Unconfined compression of the fully integrated biphasic constructs (PCL/β-TCP scaffold with GelMA/CS hydrogel in the chondral phase) yielded a compressive modulus of 5.41 ± 0.93 MPa (Figure 2E).

To further characterize the mechanical behavior beyond a single static compression measurement, cyclic compression testing was performed on representative constructs. Representative scaffold-only and hydrogel-integrated constructs exhibited reproducible hysteresis loops after the initial conditioning cycle, indicating construct-level viscoelastic energy dissipation and stable behavior under repeated compressive loading (Supplementary Figure S1). Importantly, both scaffold-only and hydrogel-integrated constructs maintained stable load–deformation behavior during cyclic compression, supporting construct-level mechanical compatibility of the hydrogel-modified design under repeated loading. Rheological analysis was further performed to characterize the upper-layer hydrogels. Flow sweep tests showed pronounced shear-thinning behavior in both GelMA and GelMA/CS hydrogels, with viscosity decreasing progressively as shear rate increased, indicating favorable processability for infiltration into the porous chondral compartment (Supplementary Figure S2). Oscillatory strain sweep further demonstrated solid-like viscoelastic behavior at low strain, where the storage modulus (G′) remained higher than the loss modulus (G″), whereas increasing strain led to progressive reduction of G′ and eventual G′/G″ crossover, consistent with strain-dependent yielding of the hydrogel network. Compared with GelMA alone, GelMA/CS maintained a broadly comparable rheological profile, indicating that CS incorporation preserved the hydrogel’s suitability for upper-layer integration while modestly modulating its viscoelastic response. Together, these results indicate that the hydrogel-modified biphasic scaffold exhibits close structural integration, stable construct-level behavior under repeated compression, and hydrogel-phase rheological properties compatible with upper-layer infiltration and incorporation.

**3.3 GelMA/CS hydrogels regulate hAC behavior**

To identify an optimal hydrogel formulation for cartilage-phase functionalization, a combinatorial matrix was prepared by pairing three GelMA concentrations (5%, 6%, and 7% w/v) with five CS concentrations (0%, 1%, 2%, 3%, and 5% w/v), yielding 15 formulations. Before direct hAC culture experiments, hydrogel formulations were preliminarily screened using hUCMSCs cultured with hydrogel-conditioned medium, and cell proliferation was assessed by CCK-8 at days 1, 3, and 5. Among the tested conditions, formulations based on 6% GelMA consistently supported favorable cell proliferation (Supplementary Figure S3A). Subsequent qRT-PCR screening focusing on 6% GelMA supplemented with 1%, 3%, or 5% CS showed that 6% GelMA/3% CS produced the most balanced upregulation of chondrogenic markers (SOX9, COL2A1, and ACAN) (Supplementary Figure S3B). This formulation was therefore selected for subsequent experiments and scaffold modification.


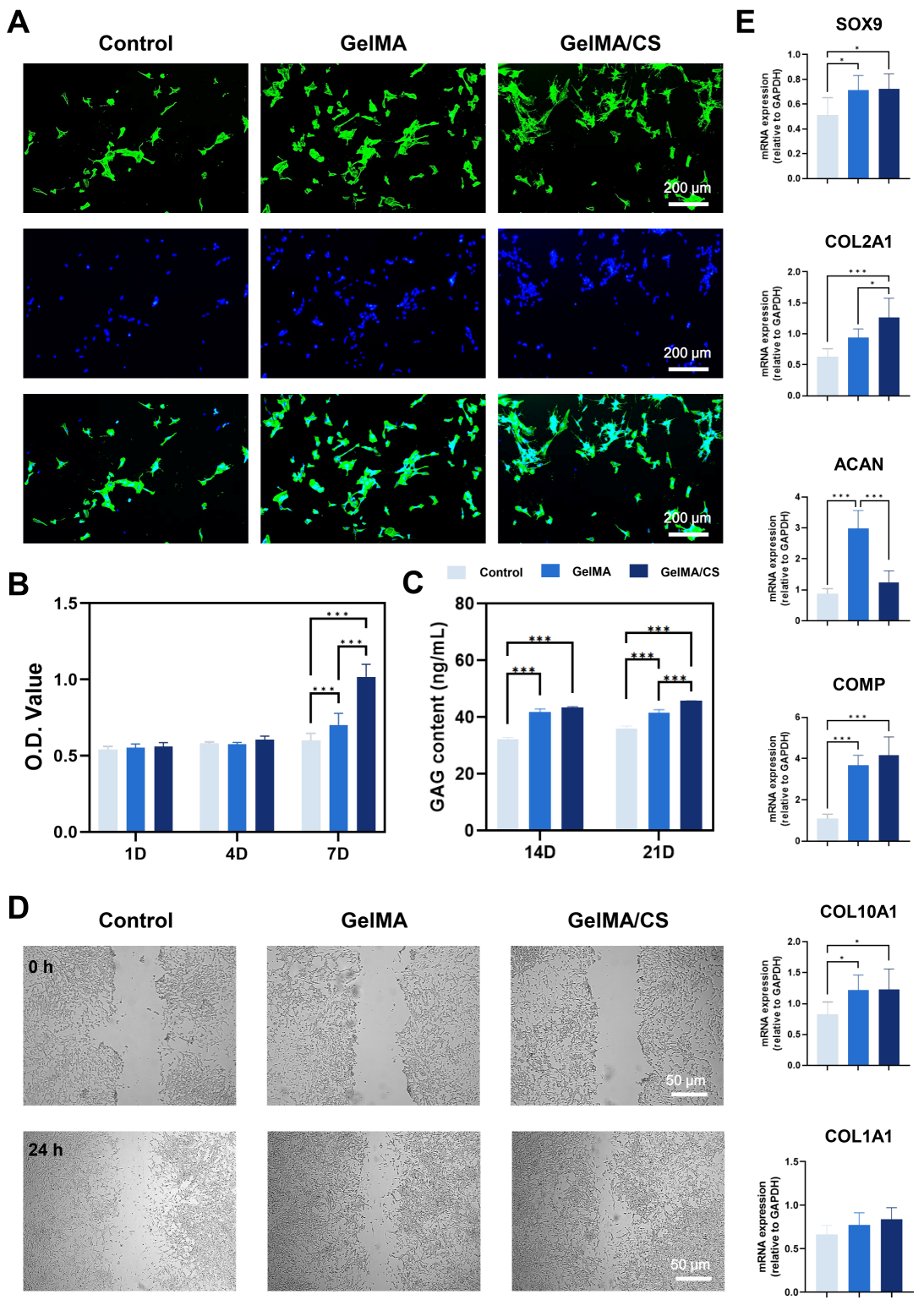


**Figure 3.** In vitro responses of human articular chondrocytes (hACs) to GelMA/CS hydrogels and hydrogel-conditioned media. (A) Confocal immunofluorescence images of hACs cultured on tissue-culture plastic control, GelMA, or GelMA/CS hydrogel surfaces, stained for F-actin and nuclei at 48 h. (B) hAC proliferation on tissue-culture plastic control, GelMA, and GelMA/CS hydrogel surfaces assessed by CCK-8 assay at days 1, 4, and 7. (C) Secreted sGAG content from hACs cultured under the same conditions, quantified by ELISA and normalized to total DNA content at days 14 and 21. (D) Scratch wound assay using control medium, GelMA extract, or GelMA/CS extract as conditioned medium; wound closure recorded at 0 and 24 h. (E) qRT-PCR analysis of chondrogenic gene expression (SOX9, COL2A1, ACAN, COMP) at day 14, normalized to GAPDH. Data are shown as mean ± SD. Statistical significance was evaluated using one-way ANOVA with Tukey’s post hoc test (*p < 0.05, **p < 0.01, ***p < 0.001).

hAC responses on hydrogel surfaces were then evaluated. F-actin staining at 48 h revealed pronounced differences in cell morphology (Figure 3A). Compared with the control group, cells cultured on GelMA and GelMA/CS hydrogels exhibited enhanced spreading with more organized cytoskeletal structures and evident cellular protrusions. Notably, the GelMA/CS group showed the largest projected cell area and the most developed F-actin network, indicating improved early adhesion/spreading on the CS-containing hydrogel (Supplementary Figure S4). Cell proliferation was assessed by CCK-8 over 7 days (Figure 3B). Both hydrogel groups showed significantly higher proliferation than the control group at days 1, 4, and 7. Moreover, GelMA/CS further increased the CCK-8 signal compared with GelMA at later time points (day 7), suggesting an additional growth-promoting effect associated with CS incorporation under these culture conditions. Cartilage-like matrix production was assessed by quantifying sGAG in culture supernatants at days 14 and 21 and normalizing to DNA content (Figure 3C). At day 14, sGAG levels were significantly elevated in both hydrogel groups compared with the control, with GelMA/CS showing the highest values. By day 21, sGAG accumulation increased across all groups, and GelMA/CS remained the highest, followed by GelMA and the control (p < 0.001), indicating sustained enhancement of matrix deposition in the presence of CS. To assess migration, scratch wound assays were performed on standard culture plates using hydrogel-conditioned media (Figure 3D and Supplementary Figure S5). GelMA/CS extracts significantly accelerated wound closure compared with GelMA extracts and control medium at 24 h, supporting a stronger pro-migratory effect of the CS-containing formulation. Chondrogenic differentiation was further evaluated by qRT-PCR at day 14 (Figure 3E). Both hydrogel groups significantly upregulated SOX9, COL2A1, and ACAN compared to the control, with GelMA/CS inducing the highest levels of SOX9 and COL2A1. ACAN expression was comparably elevated in both GelMA and GelMA/CS groups. Additionally, COMP expression was markedly increased under both conditions, suggesting enhanced ECM maturation. Expression of COL10A1 remained low across all groups, indicating minimal hypertrophic differentiation. COL1A1 showed only a slight increase with no statistically significant difference among groups, indicating that the hydrogels primarily enhanced cartilage-associated gene expression under these conditions. These results, together with the preliminary hUCMSC-based formulation screening, support the selection of 6% GelMA/3% CS as a cartilage-phase hydrogel formulation that promotes hAC adhesion/spreading, proliferation, accelerated wound closure in the scratch assay using hydrogel-conditioned media, cartilage-matrix production, and chondrogenic gene expression.


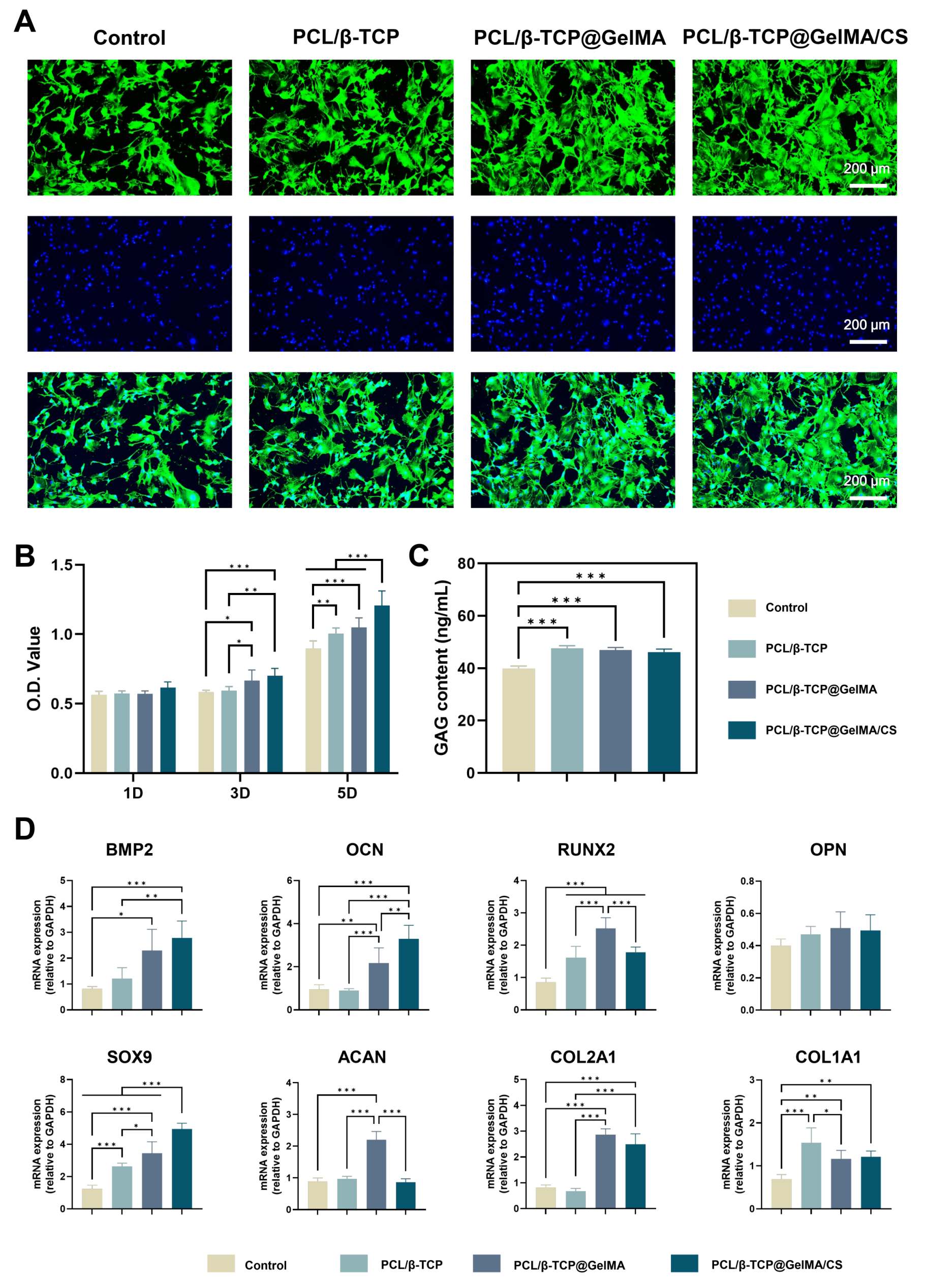


**Figure 4.** In vitro evaluation of hUCMSC proliferation and lineage-associated responses on hydrogel-functionalized biphasic scaffolds. (A) Confocal immunofluorescence images of hUCMSCs cultured on different substrates (control, uncoated biphasic PCL/β-TCP scaffold, PCL/β-TCP@GelMA scaffold, and PCL/β-TCP@GelMA/CS scaffold), stained for F-actin (phalloidin, green) and nuclei (DAPI, blue). (B) Cell proliferation assessed by CCK-8 at days 1, 3, and 5 after seeding onto the cartilage-facing (upper) region of each scaffold. (C) sGAG content at day 7, quantified by ELISA and normalized to total DNA content measured by PicoGreen. (D) qRT-PCR analysis of osteogenic markers (RUNX2, BMP2, COL1A1, OCN, OPN) and chondrogenic markers (SOX9, COL2A1, ACAN) at day 7, normalized to GAPDH. Data represent mean ± SD (n ≥ 6). Statistical analysis was performed using one-way ANOVA with Tukey’s post hoc test (*p < 0.05, **p < 0.01, ***p < 0.001).


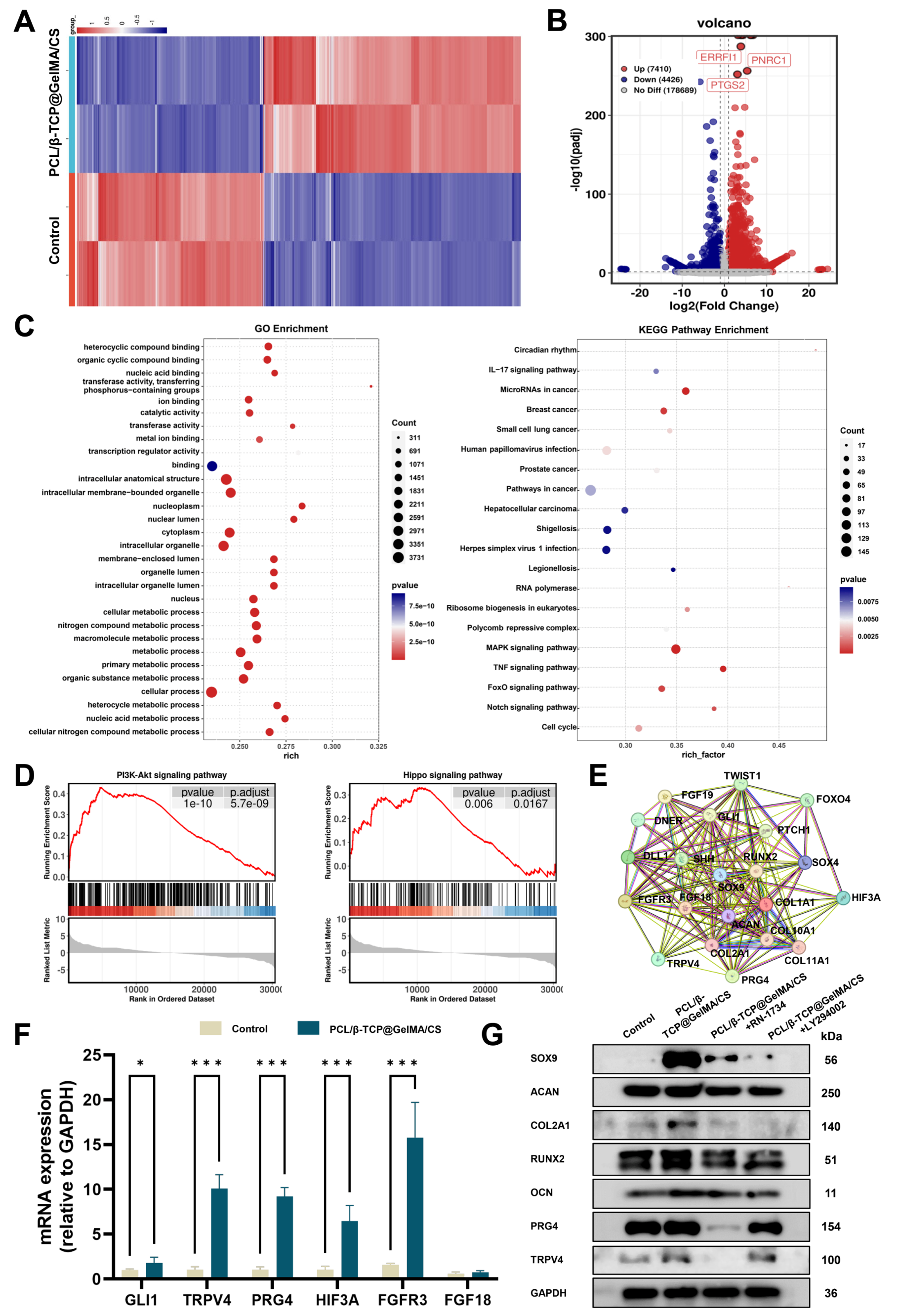


**Figure 5.** Transcriptomic profiling and inhibitor-based assessment of TRPV4- and PI3K/AKT-related signaling in hUCMSCs. Transcriptomic comparison was performed between the control group (no scaffold) and the PCL/β-TCP@GelMA/CS group after 14 days of osteochondral induction. (A) Heatmap of hierarchical clustering for representative differentially expressed genes (DEGs) across biological replicates. (B) Volcano plot of DEGs. (C) GO and KEGG pathway enrichment analysis of DEGs, highlighting biological processes and signaling pathways associated with osteochondral lineage commitment and mechanotransduction. (D) Representative gene set enrichment analysis (GSEA) plots. (E) Protein–protein interaction (PPI) network of selected DEGs, identifying hub genes related to extracellular matrix remodeling. (F) qRT-PCR validation of selected genes (GLI1, TRPV4, PRG4, HIF3A, FGFR3, and FGF18), normalized to GAPDH. (G) Western blot analysis. Inhibitor studies using RN-1734 (TRPV4) and LY294002 (PI3K) are consistent with the involvement of TRPV4-associated mechanosensing and PI3K/AKT-related signaling. Data are shown as mean ± SD. Statistical significance determined by one-way ANOVA with Tukey’s post hoc test (*p < 0.05, **p < 0.01, ***p < 0.001).

**3.4 Osteochondral differentiation of hUCMSCs on GelMA/CS-functionalized biphasic scaffolds**

To assess the capacity of hydrogel-functionalized biphasic scaffolds to direct osteochondral lineage differentiation, hUCMSCs were cultured under osteochondral induction conditions. Four experimental groups were established: (i) control (no scaffold), (ii) uncoated biphasic scaffold (PCL/β-TCP), (iii) scaffold functionalized with GelMA (PCL/β-TCP@GelMA), and (iv) scaffold functionalized with GelMA/CS (PCL/β-TCP@GelMA/CS). Representative F-actin/DAPI staining images are shown in Figure 4A. Cell proliferation was assessed using CCK-8 assays on days 1, 3, and 5 (Figure 4B). While minimal differences were observed on day 1, significant group distinctions emerged by day 3, with both hydrogel-modified scaffolds showing elevated cell proliferation compared to the uncoated scaffold and control. By day 5, the PCL/β-TCP@GelMA/CS group exhibited the highest proliferation rate, surpassing GelMA alone and indicating a sustained pro-proliferative effect attributed to CS incorporation. To evaluate cartilage-like matrix formation, sGAG production was quantified at day 7 and normalized to DNA content (Figure 4C). All scaffold-containing groups demonstrated significantly higher sGAG levels normalized to total DNA content than the scaffold-free control, indicating that the 3D scaffold environment facilitated matrix production. However, no significant differences were observed among the PCL/β-TCP, GelMA-, and GelMA/CS-functionalized scaffolds at this time point. Gene expression profiling by qRT-PCR revealed hydrogel-specific modulation of osteogenic and chondrogenic markers (Figure 4D). Among osteogenic genes, BMP2 expression was significantly upregulated in both GelMA- and GelMA/CS-modified groups compared to the control and uncoated scaffold, indicating that hydrogel incorporation broadly enhanced osteoinductive signaling. OCN, a marker of late osteogenic maturation, showed the strongest induction in the PCL/β-TCP@GelMA/CS group. Interestingly, RUNX2 expression peaked in the GelMA group, while intermediate levels were observed in the PCL/β-TCP@GelMA/CS and PCL/β-TCP groups, suggesting differential sensitivity of early osteogenic responses to hydrogel composition. For chondrogenic genes, SOX9 was markedly elevated in both hydrogel-functionalized groups, with PCL/β-TCP@GelMA/CS showing the highest expression. COL2A1 followed a similar trend, confirming enhanced cartilage-associated matrix gene activation. In contrast, ACAN showed the strongest induction in the GelMA-modified group, and CS incorporation did not further enhance ACAN at this stage. COL1A1 was elevated in all scaffold-containing groups relative to the control. Therefore, hydrogel functionalization enhanced early osteochondral gene responses, and the PCL/β-TCP@GelMA/CS group showed higher SOX9/OCN expression and stronger CCK-8 signals than the GelMA-modified or uncoated scaffold groups under the present conditions.

**3.5 Transcriptomic profiling and functional assessment of TRPV4- and PI3K/AKT-related signaling**

To investigate molecular programs associated with the cellular response to the GelMA/CS-functionalized biphasic scaffold, RNA-seq was performed on hUCMSCs cultured under osteochondral induction conditions in the control group (no scaffold) and the PCL/β-TCP@GelMA/CS group. Hierarchical clustering of representative differentially expressed genes showed clear separation between groups (Figure 5A). Differential expression analysis identified 11,836 significantly altered genes (|log₂ fold change| ≥ 1, adjusted p < 0.05), including 7,410 upregulated and 4,426 downregulated genes in the PCL/β-TCP@GelMA/CS group relative to the control group (Figure 5B), indicating a robust scaffold-associated transcriptomic shift. GO enrichment analysis indicated overrepresentation of binding-related molecular functions and intracellular structural components, reflecting broad cellular reprogramming (Figure 5C). KEGG pathway analysis revealed enrichment of several pathways associated with cellular signaling, lineage regulation, and cell–matrix interaction, including TGF-β, MAPK, Hedgehog, focal adhesion, Hippo, and ECM–receptor interaction-related pathways. In addition, multiple PI3K/AKT-related genes were represented in the transcriptomic dataset, and PI3K/AKT was therefore selected for further functional validation based on its biological relevance. Enrichment of focal adhesion and Hippo signaling further suggested a prominent role of cell–matrix mechanotransduction. GSEA corroborated these findings, revealing coordinated upregulation of gene sets linked to cartilage development, skeletal morphogenesis, and cytoskeletal organization (Figure 5D; Supplementary Figure S6). Protein–protein interaction (PPI) network analysis of hub genes highlighted key regulators including TRPV4, PRG4, HIF3A, GLI1, and growth factor-associated markers such as FGFR3 and FGF18, alongside classical osteochondral markers RUNX2, BMP2, and OCN (Figure 5E). qRT-PCR validation confirmed significant upregulation of TRPV4, PRG4, HIF3A, and FGFR3 in the PCL/β-TCP@GelMA/CS group (p < 0.001), with modest but significant elevation of GLI1 (p < 0.05), whereas FGF18 showed an upward trend without statistical significance (Figure 5F).

Western blot analysis further demonstrated increased expression of TRPV4, PRG4, and osteochondral differentiation markers (SOX9, ACAN, COL2A1, RUNX2, and OCN) in the PCL/β-TCP@GelMA/CS group compared with the control group (Figure 5G; Supplementary Figure S7). To assess the functional relevance of the identified pathways, pharmacological inhibition was performed using a TRPV4 inhibitor (RN-1734) and a PI3K inhibitor (LY294002). TRPV4 blockade markedly reduced TRPV4 protein levels and was accompanied by decreased expression of PRG4 and ACAN, as well as reduced levels of osteochondral markers including SOX9, COL2A1, RUNX2, and OCN. PI3K inhibition attenuated the expression of SOX9, COL2A1, ACAN, RUNX2, OCN, and PRG4, while showing comparatively limited effects on TRPV4. Additional phosphorylation analysis under the same inhibitor conditions showed reduced PI3K and AKT phosphorylation following TRPV4 or PI3K inhibition (Supplementary Figure S8). These inhibitor- and phosphorylation-associated changes support the involvement of TRPV4-associated mechanosensing and PI3K/AKT-related signaling in the cellular response to the GelMA/CS-functionalized scaffold.


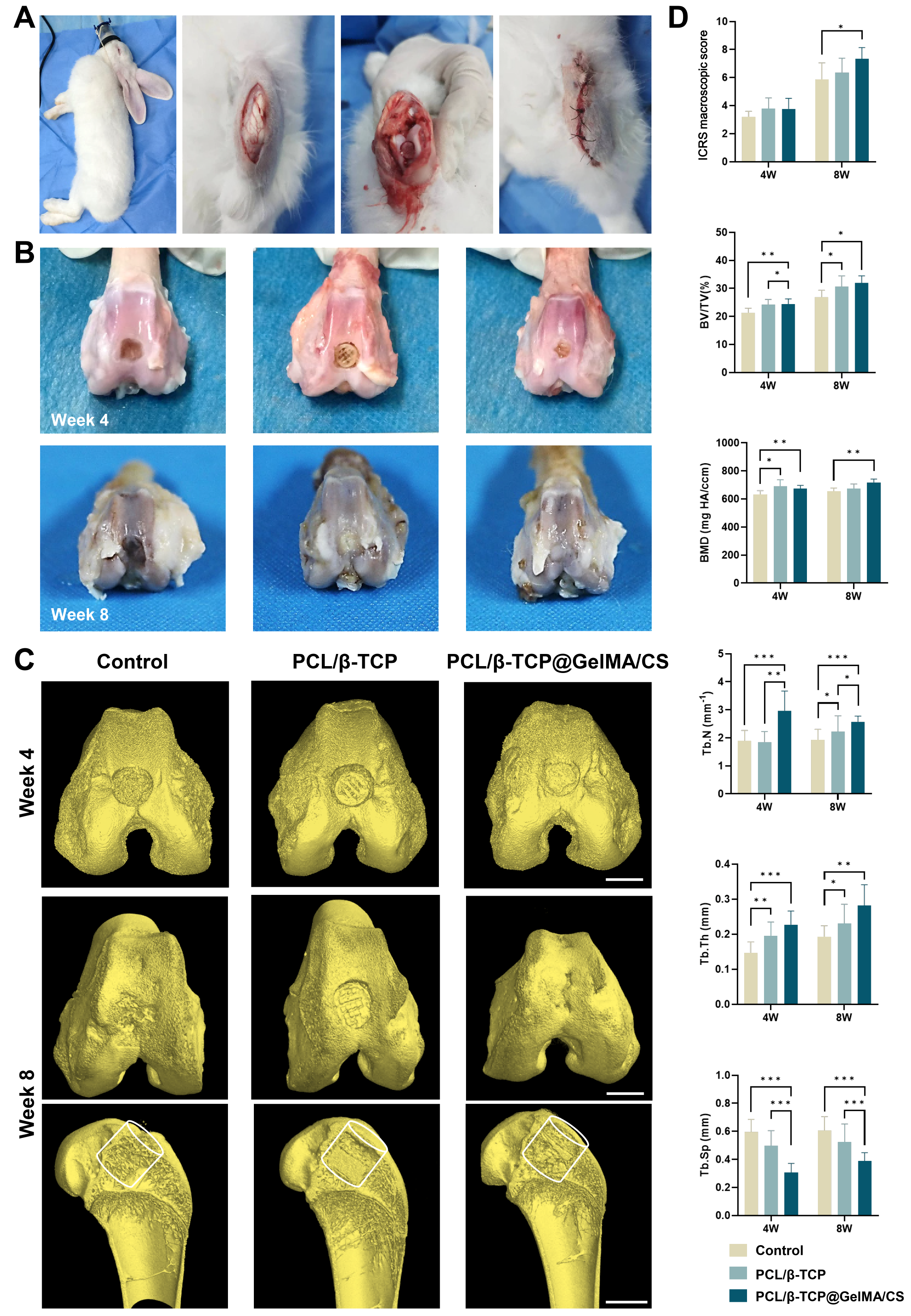


**Figure 6.** In vivo evaluation of osteochondral repair in a rabbit trochlear defect model. (A) Schematic of the surgical procedure and experimental design. Bilateral full-thickness cylindrical defects (Ø 5 mm × 5 mm depth) were created in the femoral trochlear grooves of rabbits. Three treatment conditions were included: control (no implant), unmodified PCL/β-TCP biphasic scaffold, and GelMA/CS-functionalized PCL/β-TCP scaffold (PCL/β-TCP@GelMA/CS). Treatments were assigned in a randomized bilateral paired manner such that each rabbit received two different treatments, one per knee. (B) Representative macroscopic images of distal femurs at 4 and 8 weeks post-implantation. (C) Micro-CT 3D reconstructions and sagittal cross-sections of the defect sites at each time point. (D) Quantitative analysis including ICRS macroscopic scores and micro-CT parameters: bone volume fraction (BV/TV), bone mineral density (BMD), trabecular number (Tb.N), trabecular thickness (Tb.Th), and trabecular separation (Tb.Sp). Data are presented as mean ± SD. At each time point, 9 rabbits (18 knees) were included, with 6 knees represented in each treatment group. In vivo data were analyzed using a linear mixed-effects model to account for bilateral pairing within each animal (*p < 0.05, **p < 0.01, ***p < 0.001).

**3.6 Micro-CT evaluation of osteochondral regeneration**

In vivo osteochondral repair was evaluated in full-thickness cylindrical defects (5 mm diameter × 5 mm depth) created in the femoral trochlear grooves of New Zealand white rabbits (Figure 6A). Defects were left untreated (control) or implanted with an uncoated biphasic PCL/β-TCP scaffold or a GelMA/CS-functionalized biphasic scaffold (PCL/β-TCP@GelMA/CS). All animals recovered from surgery without obvious complications, and no adverse reactions, wound dehiscence, or wound-healing disorders were observed during the observation period. Gross observation at 4 and 8 weeks revealed time-dependent differences in defect filling and surface restoration (Figure 6B). At 4 weeks, the control group exhibited a persistent circular depression with minimal tissue coverage. The uncoated PCL/β-TCP group retained a clearly visible porous framework at the surface, indicating limited early surface remodeling. In contrast, the PCL/β-TCP@GelMA/CS group showed a smaller apparent defect opening with partial surface coverage and improved integration with adjacent cartilage. By 8 weeks, the control group still displayed an obvious depression and incomplete repair. The uncoated scaffold group achieved partial surface closure but often with uneven topography. Notably, the PCL/β-TCP@GelMA/CS group presented a smoother, more continuous white, cartilage-like surface with improved defect filling and margin integration, approaching the surrounding native cartilage in gross appearance.

Micro-CT was used to assess subchondral bone regeneration within the defect region (Figure 6C). At 4 weeks, the control group showed a persistent void with negligible mineralized tissue. The uncoated PCL/β-TCP group maintained a well-defined interconnected porous architecture in the subchondral region, with early trabecular ingrowth apparent in deeper zones. The PCL/β-TCP@GelMA/CS group exhibited reduced void space and less sharply delineated internal scaffold features, consistent with increased tissue infiltration and remodeling within the defect volume. At 8 weeks, sagittal reconstructions showed progressive subchondral repair extending from the host bone margins in all groups. The uncoated scaffold group typically retained discernible remnants in the deeper zone accompanied by new bone formation, whereas the PCL/β-TCP@GelMA/CS group displayed more continuous mineralized tissue throughout the defect depth with less apparent residual scaffold signal. Across groups, radiopaque signals in the superficial region remained limited, which is expected because the upper cartilage phase is composed of radiolucent PCL and is designed to support non-mineralized cartilage formation, resulting in low micro-CT contrast in the top layer.

Consistent with gross findings, ICRS macroscopic scores showed no significant differences among groups at 4 weeks, whereas at 8 weeks the PCL/β-TCP@GelMA/CS group achieved significantly higher scores than the control group, reflecting improved surface regularity, defect filling, and integration. Quantitative micro-CT analysis of subchondral bone within the defined VOI further supported scaffold-associated improvements (Figure 6D). At 4 weeks, both scaffold-treated groups exhibited higher BV/TV and BMD than the control group, with PCL/β-TCP@GelMA/CS showing the highest values. This trend remained at 8 weeks, particularly for BMD in the PCL/β-TCP@GelMA/CS group. Trabecular microarchitecture parameters were also improved in scaffold-treated defects, with higher Tb.N and Tb.Th and lower Tb.Sp relative to the control group, indicating a shift toward a denser trabecular structure. These results indicate that the GelMA/CS-functionalized scaffold was associated with improved early defect filling and enhanced subchondral bone regeneration within the current observation period compared with the uncoated scaffold and untreated control. However, because an in vivo GelMA-only group was not included, the present animal data do not allow definitive isolation of the incremental contribution of CS relative to GelMA alone. In addition, micro-CT assessment of the superficial cartilage phase is inherently limited by the radiolucency of the PCL-based upper layer.

**3.7 Histological evaluation of osteochondral regeneration in vivo**

Histological analysis of femoral specimens harvested at 4 and 8 weeks was performed using H&E, Masson’s trichrome, Toluidine Blue (TB), and Safranin O/Fast Green (SO/FG) staining to evaluate tissue regeneration, scaffold–host integration, cellular infiltration, collagen-rich matrix organization, and GAG-rich cartilage ECM deposition (Figure 7). At 4 weeks, the control group (untreated defect) displayed persistent voids predominantly filled with fibrovascular tissue, with limited organized tissue restoration. Adjacent to the host bone margin, clusters of hypertrophic chondrocyte-like cells were observed and appeared as localized purple-stained interfaces in Masson’s and TB staining, suggesting an endochondral-like reparative response at the bone interface. These cells were embedded within immature matrix, indicating an early but disorganized repair pattern. In the uncoated PCL/β-TCP scaffold group, partial subchondral bone ingrowth extended from the host margins into the β-TCP-containing porous phase. H&E and Masson’s trichrome staining showed osteoid-like matrix deposition and moderate collagen accumulation at the defect base, consistent with early osteogenic remodeling. However, the upper PCL layer remained largely unfilled, with sparse TB and SO/FG staining, indicating limited chondral tissue regeneration. In contrast, the PCL/β-TCP@GelMA/CS group exhibited more extensive early regeneration. The lower β-TCP phase showed denser cellular infiltration and more apparent new bone formation within scaffold pores. In the superficial region, TB and SO/FG staining already demonstrated patchy but clearly positive GAG-associated signals, indicating earlier initiation of cartilage-like ECM deposition compared with the other groups.

By 8 weeks, intergroup differences became more evident. The control group continued to show incomplete healing with irregular defect margins, persistent fibrous tissue, and limited cartilage restoration, while hypertrophic chondrocyte-like cells remained visible near the bone interface. The uncoated PCL/β-TCP group exhibited further maturation of subchondral bone with trabecular structures partially integrating with host tissue; nevertheless, the superficial region showed poor surface continuity and weak GAG staining, indicating limited chondral repair despite subchondral improvement. The PCL/β-TCP@GelMA/CS group displayed the most advanced osteochondral restoration. The β-TCP-containing phase was substantially replaced by newly formed bone with a more organized architecture, and the superficial layer exhibited more continuous GAG-rich matrix with stronger TB and SO/FG staining approaching the staining intensity and distribution of native cartilage. A tidemark-like boundary was observed between the regenerated cartilage and underlying bone, suggesting formation of a stratified osteochondral interface. Masson’s trichrome further indicated improved collagen continuity and matrix organization across the repair region. In some specimens, the regenerated cartilage surface appeared slightly recessed relative to the surrounding tissue, forming a narrow surface depression, suggesting that surface contouring and remodeling may still be ongoing at this time point. In addition, gross observation at specimen harvest and routine histological assessment of the defect region did not reveal obvious abnormal local swelling or marked inflammatory reactions. To preliminarily evaluate the in vivo biosafety of the implanted scaffolds, major organs including the heart, liver, spleen, lung, and kidney were harvested at the experimental endpoint and subjected to H&E staining. No obvious pathological abnormalities, including marked inflammatory cell infiltration, tissue necrosis, or structural damage, were observed in the examined organs, suggesting acceptable short-term systemic biocompatibility of the implanted constructs (Supplementary Figure S9). Overall, histological analyses indicated that GelMA/CS functionalization was associated with enhanced early cellular infiltration, improved subchondral bone regeneration, and more continuous cartilage-like matrix formation by 8 weeks, consistent with the gross observations, ICRS scoring, and micro-CT findings.


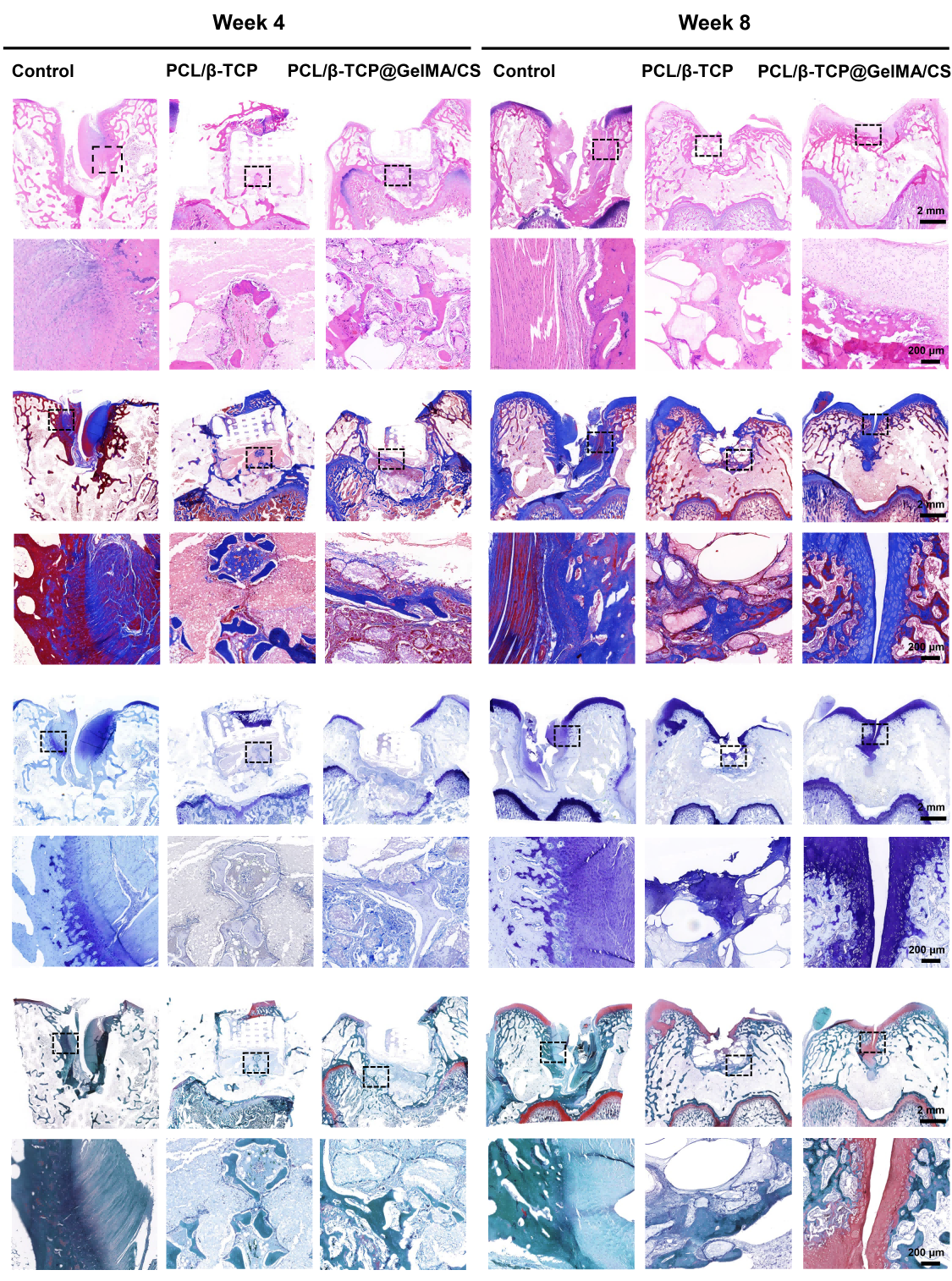


**Figure 7.** Histological analysis of osteochondral regeneration in vivo. Representative histological staining of femoral trochlear defects harvested at 4 and 8 weeks post-implantation across all groups (control, unmodified PCL/β-TCP, and PCL/β-TCP@GelMA/CS). Sections were stained with Hematoxylin–Eosin (H&E), Masson’s trichrome, Toluidine Blue (TB), and Safranin O/Fast Green (SO/FG) to evaluate overall tissue morphology, collagen organization, and cartilage matrix (GAG-rich) deposition. The black dashed boxes in the upper panels indicate the regions shown at higher magnification in the corresponding lower panels.


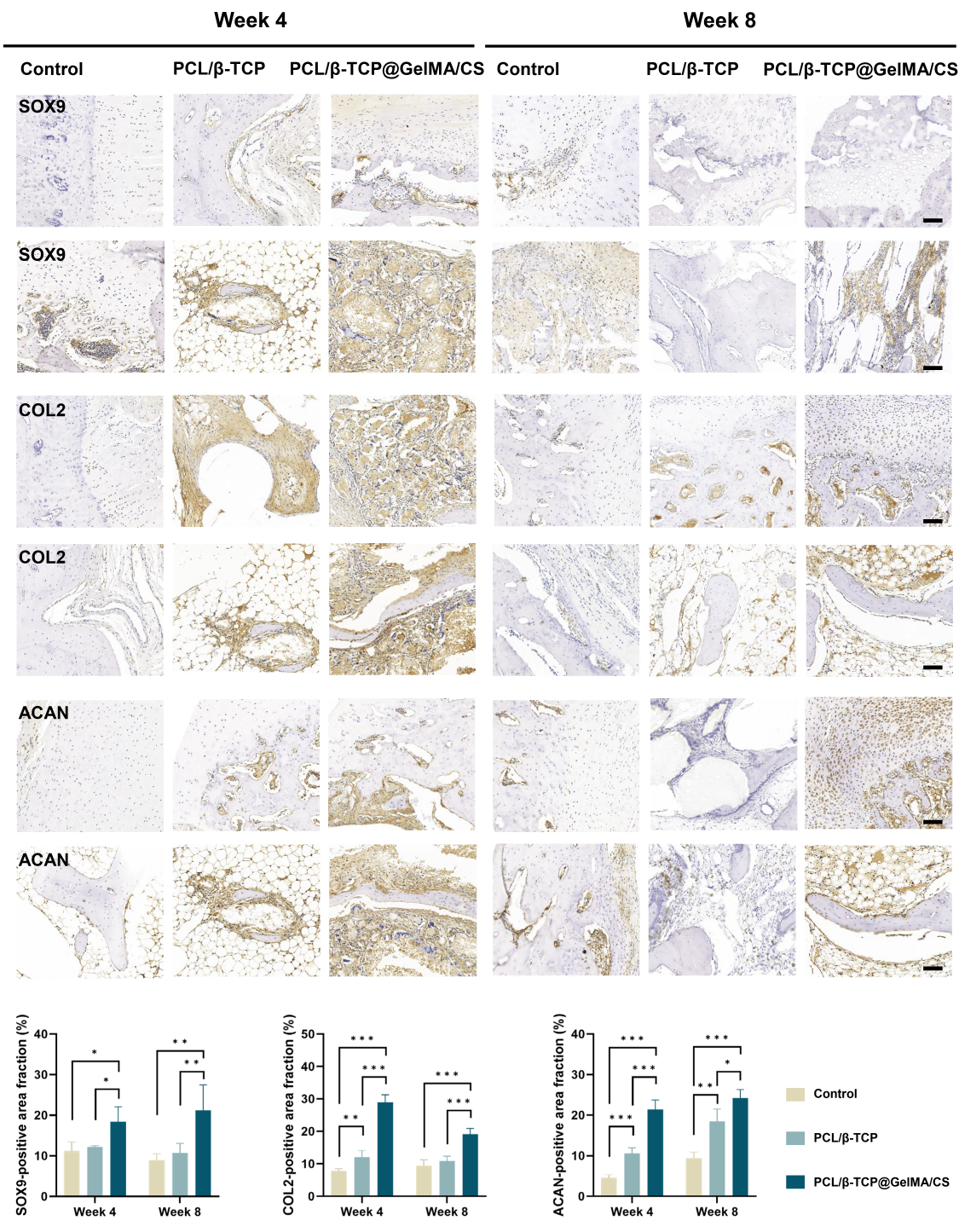


**Figure 8.** Immunohistochemical assessment of chondrogenic marker expression. Representative immunohistochemical staining of SOX9, type II collagen (COL2), and aggrecan (ACAN) in rabbit trochlear defects at 4 and 8 weeks post-implantation. Images are shown at 20× magnification. Representative images include superficial and deep repair regions to illustrate the spatial distribution of marker expression. Semi-quantitative analysis was performed as DAB-positive area fraction (%) within predefined regions of interest (ROIs). Data are presented as mean ± SD. At each time point, 9 rabbits (18 knees) were included, with 6 knees per treatment group. In vivo data were reanalyzed using a linear mixed-effects model to account for bilateral pairing within each animal (*p < 0.05, **p < 0.01, ***p < 0.001).

**3.8 Immunohistochemical evaluation of chondrogenic markers**

Immunohistochemical (IHC) staining was performed on decalcified paraffin sections harvested at 4 and 8 weeks post-implantation to examine protein-level expression of chondrogenic markers, including SOX9, COL2, and ACAN (aggrecan) (Figure 8). Whole-slide images were digitally scanned using a CaseViewer system, and representative 20× fields were selected from the superficial (cartilage) and deep (subchondral) zones using consistent anatomical landmarks. At 4 weeks, the control group showed minimal SOX9 staining in the superficial region, with weak signals in deep-zone repair tissue. The uncoated scaffold group (PCL/β-TCP) exhibited similarly low-level SOX9 positivity, mainly localized to peri-implant repair regions in the deep zone. In contrast, the hydrogel-functionalized scaffold group (PCL/β-TCP@GelMA/CS) displayed more evident SOX9-positive cells in the deep-zone repair tissue, frequently distributed around scaffold-associated regions. In the superficial zone, SOX9-positive cells were detectable but remained weak across groups. By 8 weeks, SOX9 staining generally decreased in all groups, while the PCL/β-TCP@GelMA/CS group retained discernible SOX9 positivity in deep-zone repair regions. COL2 staining was very limited in the control group at 4 weeks in both zones. The PCL/β-TCP group showed detectable COL2 positivity in peri-implant repair tissue. Notably, the PCL/β-TCP@GelMA/CS group exhibited stronger and more continuous COL2 staining in both compartments, including distinct signals within cartilage-like structures in the superficial layer. At 8 weeks, COL2 positivity remained broader in the PCL/β-TCP@GelMA/CS group, spanning the superficial neotissue and subchondral repair regions, whereas the PCL/β-TCP group showed moderate and more discontinuous staining; the control group remained weakly positive. ACAN expression followed a similar trend. At 4 weeks, the control and PCL/β-TCP groups exhibited weak superficial ACAN staining with modest signals in the deep zone. The PCL/β-TCP@GelMA/CS group showed more apparent ACAN positivity in both zones, with localized staining in newly formed cartilage-like tissue. By 8 weeks, ACAN staining remained more consistently detectable in the PCL/β-TCP@GelMA/CS group in both the superficial neotissue and deep-zone repair regions, while signals in the other groups were comparatively reduced. Semi-quantitative analysis of DAB-positive area fraction within predefined ROIs supported these observations. Overall, the PCL/β-TCP@GelMA/CS group generally exhibited stronger staining for SOX9, COL2, and ACAN than the PCL/β-TCP and control groups at both time points, consistent with enhanced cartilage-associated matrix formation during early repair.

**4. Discussion**

Osteochondral defects continue to pose a major clinical challenge because the native osteochondral unit is highly zonal in composition, microstructure, and mechanical function, whereas articular cartilage has minimal intrinsic healing capacity due to its avascular and hypocellular nature [29, 30]. Current clinical options, including microfracture and osteochondral autograft/allograft transplantation, may provide short-term symptom relief but frequently lead to fibrocartilage formation, suboptimal cartilage–bone integration, and compromised long-term durability [31]. Accordingly, a successful tissue-engineered approach should address two interdependent requirements: (i) re-establishing a mechanically continuous load-transfer pathway across the cartilage–bone interface, and (ii) providing a cartilage-permissive microenvironment that supports chondrogenic matrix deposition and helps preserve (or induce) a stable chondrogenic phenotype [32, 33]. Multilayered or gradient scaffolds are conceptually attractive because they recapitulate osteochondral hierarchy and enable spatial coordination of osteogenic and chondrogenic cues [34]. However, accumulating evidence suggests that stratification alone—whether by material composition or pore architecture—does not necessarily ensure functional integration, particularly under joint loading where abrupt mechanical discontinuities and weak interfacial bonding can promote micro-motion and stress concentration, ultimately undermining repair stability and durability [35-37]. These considerations highlight that effective osteochondral regeneration requires not only zoned biological signaling but also interfacial mechanical coherence across phases—an engineering balance that remains challenging in many current scaffold designs.

Building on this rationale, we developed a structurally continuous yet functionally stratified biphasic scaffold that integrates a β-TCP-reinforced PCL subchondral compartment with a cartilage-guiding pure PCL upper layer within a single 3D-printed architecture. This design was driven by the need to reconcile biological compartmentalization with structural continuity and mechanical compatibility, two attributes that are frequently decoupled in conventional multilayer scaffolds. Although trilayer constructs incorporating a calcified-cartilage-like intermediate zone have demonstrated improved cue segregation and barrier-type behavior in preclinical models, they often rely on multi-step fabrication (e.g., discrete isolation layers, separately processed modules, or post-assembly lamination) [38–41]. Such complexity increases manufacturing burden, may introduce potential interfacial weak points under repeated loading, and can hinder scalability for translation. In contrast, our approach employs a sequential dual-temperature extrusion-based FDM workflow to achieve continuous interlayer fusion while establishing zone-specific pore architectures: large macropores in the β-TCP-enriched subchondral phase to support vascularized bone ingrowth, and smaller pores in the upper PCL layer to facilitate cell retention and cartilage-like matrix deposition. Instead of incorporating a discrete interface layer, we applied a top-down infiltration of GelMA/CS hydrogel into the upper ~1.5 mm of the construct, while intentionally preserving a ~0.5 mm hydrogel-free PCL transition zone. This configuration was designed to spatially confine the hydrogel within the chondral compartment while maintaining a hydrogel-free PCL region between the hydrogel-filled and β-TCP-containing phases. Notably, this “printed scaffold + localized hydrogel” approach provides a scalable and modular platform: the load-bearing architecture is produced in an integrated printing step, while the bioactive microenvironment can be tuned through hydrogel composition and spatial placement without redesigning the entire construct. Consistent with this design intent, our in vitro and rabbit osteochondral defect results indicate that the functionalized scaffold supports cartilage-associated matrix formation in the superficial region while enhancing subchondral bone regeneration, thereby supporting more coordinated early osteochondral repair.

Beyond macroscopic and histological outcomes, the enhanced osteochondral regeneration observed in the GelMA/CS-functionalized scaffolds was further supported by transcriptomic and functional analyses, which implicated key mechanosensitive and microenvironment-responsive pathways in mediating zone-specific differentiation. Among these, TRPV4, a mechanosensitive calcium channel known to regulate cartilage homeostasis by transducing mechanical and osmotic cues into intracellular signaling, emerged as a biologically meaningful regulator in the context of the present scaffold design [42–44]. The combination of the chondroitin sulfate-enriched hydrogel and scaffold pore architecture likely provided a biomechanically active environment associated with TRPV4-related signaling. Consistently, pharmacologic TRPV4 inhibition suppressed both chondrogenic markers (SOX9, COL2A1, and ACAN) and osteogenic markers (RUNX2 and OCN), supporting its involvement in dual-lineage regulation. PI3K/AKT-related signaling was also implicated by transcriptomic clues, phosphorylation analysis, and functional inhibition, all of which were consistent with reduced osteochondral marker expression under pathway inhibition. These observations are in line with prior evidence that PI3K/AKT acts as an important regulatory node linking microenvironmental stimuli with matrix synthesis, cell survival, and lineage-specific differentiation in cartilage and bone contexts [45–47]. Within the limits of the present pharmacological inhibition setup, our data support the involvement of both TRPV4-associated mechanosensing and PI3K/AKT-related signaling in the response to the functionalized scaffold, without resolving their strict hierarchical relationship or relative dominance. In addition to PI3K/AKT-related programs, transcriptomic enrichment and network-level analysis also implicated other biologically relevant signals. GLI1 showed a significant increase in our targeted validation, consistent with engagement of a broader osteochondral patterning/remodeling program [48]. PRG4, a superficial-zone-associated molecule with boundary-lubricating and anti-inflammatory relevance in cartilage, also showed increased expression and sensitivity to pathway inhibition, although the protein-level changes remained modest [49,50]. HIF3A was likewise elevated in the functionalized scaffold condition; given its reported association with suppression of hypertrophic progression, this finding is consistent with maintenance of a more stable, non-hypertrophic chondrogenic phenotype [51]. These findings are consistent with a model in which TRPV4-associated mechanosensing and PI3K/AKT-related signaling contribute to scaffold-guided osteochondral lineage specification, while additional signals such as GLI1 and PRG4 may help maintain zonal fidelity and phenotypic stability.

The in vivo results highlight the importance of combining scaffold architecture with localized biochemical functionalization to support coordinated osteochondral repair. Untreated defects predominantly healed through fibrovascular tissue infiltration, with minimal restoration of cartilage or subchondral bone structure, consistent with the limited spontaneous repair capacity of full-thickness osteochondral injuries [41,52]. The unmodified biphasic PCL/β-TCP scaffold supported partial subchondral bone ingrowth and modest neotissue formation, yet it did not consistently re-establish a smooth articular surface or clear zonal organization. This outcome reflects a common limitation of conventional biphasic designs, in which insufficient instructive cues at the chondral–osseous interface can lead to discontinuous tissue formation and/or fibrotic bridging across layers [53]. In contrast, the PCL/β-TCP@GelMA/CS constructs achieved spatially compartmentalized yet functionally integrated repair. The β-TCP-enriched basal layer supported subchondral bone regeneration accompanied by progressive scaffold remodeling, whereas the hydrogel-confined upper region favored cartilage-associated matrix deposition and helped restrict osseous ingrowth into the chondral compartment. Notably, this zonal fidelity was attained without a discrete calcified barrier layer, suggesting that controlled hydrogel confinement together with porosity gradients may partially recapitulate key features of the native osteochondral transition. Histological staining and micro-CT analysis corroborated this coordinated response, revealing more advanced subchondral bone regeneration and remodeling, while immunohistochemistry confirmed stronger expression of chondrogenic markers (SOX9, COL2A1, and ACAN) in the superficial neotissue. An additional design consideration is the non-mineralized, radiolucent superficial PCL phase, which helped preserve a cartilage-permissive compartment and was consistent with the limited radiopaque signals observed in the upper layer by micro-CT. However, the relatively slow degradation of PCL, particularly in the superficial compartment, may constrain longer-term tissue remodeling and complete replacement by native tissue. Therefore, the present 4- and 8-week findings should be interpreted as evidence of early-stage osteochondral repair rather than definitive long-term regeneration. Longer-term follow-up will be required to assess tissue maturation, scaffold remodeling, and repair stability. Taken together, these findings suggest that combining a high-ceramic, bone-mimetic base with a spatially restricted GelMA/CS-functionalized hydrogel region and a transitional hydrogel-free zone supports coordinated early osteochondral repair under the present experimental conditions. Together with transcriptomic and inhibitor-based data suggesting the involvement of TRPV4-associated mechanosensing and PI3K/AKT-related signaling, this modular platform offers a scalable and translationally relevant strategy for osteochondral defect repair.

**5. Conclusion**

In this study, we developed a structurally continuous yet functionally compartmentalized biphasic scaffold by integrating a 3D-printed PCL/β-TCP subchondral framework with a spatially confined GelMA/CS hydrogel cartilage phase. This strategy enabled region-specific modulation of pore architecture, material composition, and biochemical cues, resulting in a structurally continuous scaffold capable of supporting distinct chondral and osseous microenvironments. In vitro experiments demonstrated that the GelMA/CS-functionalized upper region promoted a cartilage-favorable response, characterized by enhanced expression of SOX9, COL2A1, and ACAN, alongside improved matrix deposition. The β-TCP-enriched basal layer maintained osteoconductive potential, supporting osteogenic marker expression and mineral-associated features. In vivo, the functionalized scaffold was associated with improved early repair outcomes compared with the unmodified scaffold and untreated controls, as evidenced by improved macroscopic morphology, micro-CT parameters of subchondral bone, and histological organization. Immunohistochemical staining further supported stronger cartilage-like matrix regeneration in the GelMA/CS group. Importantly, this coordinated osteochondral repair was achieved without the use of a discrete calcified interface, highlighting the effectiveness of spatial microenvironment engineering through a “printed scaffold + localized hydrogel” approach. This design balances structural simplicity with biological functionality, offering a scalable, modular, and translationally relevant platform for osteochondral defect repair.

**Acknowledgements**

F.Z. and X.Y.P. contributed equally to this work. The authors express gratitude to Dr. Li Chen from the Analytical & Testing Center of Sichuan University for assistance with micro-CT imaging.

**Funding**

This work was supported by the Medical Key Strategic Project of Wuxi Health Commission and the Zhenjiang City Science and Technology Project (Social Development) (Grant No. SH2024043).

**Keywords**

Osteochondral defect, biphasic scaffold, 3D printing, TRPV4, PI3K–AKT signaling

**Conflicts of interest**

Xiao Yang, Xiangdong Zhu, and Xingdong Zhang serve on the editorial board of Regenerative Biomaterials. They were not involved in the editorial handling, peer review, or decision-making process for this manuscript. The other authors declare that they have no competing interests.

**References**

1. Li F, Yang X, Chen Y, Gong M, Li L, Chen A, Dan N, Li Z. Bionic gradient scaffolds for osteochondral tissue engineering: construction strategies, interface optimization, gradient characterization, and controllability research. *Biomaterials Science* 2026; 14:305-339.

2. Liu W, Guo NY, Wang JQ, Xu BB. Osteoarthritis: mechanisms and therapeutic advances. *MedComm* 2025;6:e70290.

3. Talouki PY, Tamimi R, Rudi SG. A comprehensive review of curcumin-based scaffolds in cartilage tissue engineering. *Stem Cell Research & Therapy* 2025;16:528.

4. Xu X, Gao J, Liu S, Chen L, Chen M, Yu X, Ma N, Zhang J, Chen X, Zhong L, Yu L, Xu L, Guo Q, Ding J. Magnetic resonance imaging for non-invasive clinical evaluation of normal and regenerated cartilage. *Regenerative Biomaterials* 2021;8:rbab038.

5. Lesage C, Lafont M, Guihard P, Weiss P, Guicheux J, Delplace V. Material‐assisted strategies for osteochondral defect repair. *Advanced Science* 2022;9:2200050.

6. Gao J, Ding X, Yu X, Chen X, Zhang X, Cui S, Shi J, Chen J, Yu L, Chen S, Ding J. Cell-Free Bilayered Porous Scaffolds for Osteochondral Regeneration Fabricated by Continuous 3D-Printing Using Nascent Physical Hydrogel as Ink. *Advanced Healthcare Materials* 2021;10:2001404.

7. Deng W, Wang T, Li L, Xiao X, Xu Y, Li Q, Zhou Q, Yin Y, Yang H, Gong K. A review of nanomaterials in osteoarthritis treatment and immune modulation. *Regenerative Biomaterials* 2025:rbaf048.

8. Li C, Zhang W, Nie Y, Jiang D, Jia J, Zhang W, Li L, Yao Z, Qin L, Lai Y. Integrated and Bifunctional Bilayer 3D Printing Scaffold for Osteochondral Defect Repair. *Advanced Functional Materials* 2023;33:2214158.

9. Heydarigoojani M, Farokhi M, Simorgh S. Bioinks for engineering gradient-based osteochondral and meniscal tissue substitutes: a review. *Biofabrication* 2025; 17(2):adb0f4..

10. Yilmaz H, Abdulazez IF, Gursoy S, Kazancioglu Y, Ustundag CB. Cartilage Tissue Engineering in Multilayer Tissue Regeneration. *Annals of Biomedical Engineering* 2025;53:284-317.

11. Liu G, Wei X, Zhai Y, Zhang J, Li J, Zhao Z, Guan T, Zhao D. 3D printed osteochondral scaffolds: design strategies, present applications and future perspectives. *Frontiers in bioengineering and biotechnology* 2024;12:1339916.

12. Silva B, Domingos M, Amado S, R. Dias J, Pascoal-Faria P, Maurício AC, Alves N. Toward Integrative Biomechanical Models of Osteochondral Tissues: A Multilayered Perspective. *Bioengineering* 2025;12:649.

13. Idumah CI. Poly (α-caprolactone)(PCL) biopolymeric bionanoarchitectures for tissue engineering applications. *International Journal of Polymeric Materials and Polymeric Biomaterials* 2025;74:733-762.

14. Zhou S, Xing J, Sun L, Hu X, Liu T, Liu Q, Zhang Z, Hao M, Liang B, Xing D. Melt electrowriting: a promising 3D printing technology for cartilage and osteochondral repair. *Advanced Therapeutics* 2024;7:2300218.

15. Peng S, Hao Y, Zhou H, Chen Y, Chen M. Rational Structure Design‐Based 3D‐Printed Cardiovascular Implants. *Advanced Healthcare Materials* 2025:e04628.

16. Ni X, Feng J, Liang M, Zhou F, Xia Y, Dong Z, Xue Q, Li Z, Pu F, Xia P. Enhancing bone repair with β-TCP-based composite scaffolds: a review of design strategies and biological mechanisms. *Orthopedic Research and Reviews* 2025:313-340.

17. Zhao R, Meng X, Pan Z, Li Y, Qian H, Zhu X, Yang X, Zhang X. Advancements in nanohydroxyapatite: synthesis, biomedical applications and composite developments. *Regenerative Biomaterials* 2025;12:rbae129.

18. Barzanouni H, Houreh AB, Solati-Hashjin M, Barzanouni A, Shafieian M. 3D-Printed PLA:β-TCP Scaffolds: Fabrication and Evaluation for Bone Regeneration in Load-Bearing Defects. *Polymers for Advanced Technologies* 2025;36:e70466.

19. Tandon B, Sabahi N, Farsi R, Kangur T, Boero G, Bertsch A, Li X, Brugger J. Performance Comparison of Shape Memory Polymer Structures Printed by Fused Deposition Modeling and Melt Electrowriting. *Advanced Materials Technologies* 2025;10:2400466.

20. Li B, Ma Y, Fatima K, Zhou X, Chen S, He C. 3D printed scaffolds with multistage osteogenic activity for bone defect repair. *Regenerative Biomaterials* 2025;12:rbaf010.

21. Patel KD, Lamarra KA, Sawadkar P, Ludwig A, Perriman AW. Silk Fibroin/GelMA-Based Hydrogels as a Platform for Tissue Adhesives and Tissue Engineering. *ACS Biomaterials Science & Engineering* 2025.

22. Liu C, Yu Q, Yuan Z, Guo Q, Liao X, Han F, Feng T, Liu G, Zhao R, Zhu Z. Engineering the viscoelasticity of gelatin methacryloyl (GelMA) hydrogels via small “dynamic bridges” to regulate BMSC behaviors for osteochondral regeneration. *Bioactive Materials* 2023;25:445-459.

23. Behan K, Dufour A, Garcia O, Kelly D. Methacrylated cartilage ECM-based hydrogels as injectables and bioinks for cartilage tissue engineering. *Biomolecules* 2022;12:216.

24. Martyniak K, Lokshina A, Cruz MA, Karimzadeh M, Kemp R, Kean TJ. Biomaterial composition and stiffness as decisive properties of 3D bioprinted constructs for type II collagen stimulation. *Acta Biomaterialia* 2022;152:221-234.

25. Murphy CA, Serafin A, Collins MN. Development of 3D printable gelatin methacryloyl/chondroitin sulfate/hyaluronic acid hydrogels as implantable scaffolds. *Polymers* 2024;16:1958.

26. Ai C, Liu L, Wong K, Tan XH, Goh JC. The effect of chondroitin sulfate concentration and matrix stiffness on chondrogenic differentiation of mesenchymal stem cells. *Biomaterials Science* 2023;11:4557-4573.

27. Ke D, Yu J, Liu P, Niu C, Yang X. Biomimetic 3D printed PCL/TCP/GelMA scaffolds with improved osteogenesis and angiogenesis for non-load bearing applications. *Materialia* 2022;21:101339.

28. He X, He S, Xiang G, Deng L, Zhang H, Wang Y, Li J, Lu H. Precise lubrication and protection of cartilage damage by targeting hydrogel microsphere. *Advanced Materials* 2024;36:2405943.

29. Wu H, Wang X, Wang G, Yuan G, Jia W, Tian L, Zheng Y, Ding W, Pei J. Advancing Scaffold‐Assisted Modality for In Situ Osteochondral Regeneration: A Shift From Biodegradable to Bioadaptable. *Advanced Materials* 2024;36:2407040.

30. Stocco E, Confalonieri M, Barbon S, Frison C, Acquasaliente L, Boscolo-Pecchie R, Toro Marin V, Contran M, Boscolo-Berto R, Brun P. The impact of microstructure and extracellular matrix suspension on the proliferation of bone marrow-derived mesenchymal stem cells for osteochondral defect repair. *Regenerative Biomaterials* 2025;12:rbaf109.

31. Choe R, Devoy E, Jabari E, Packer JD, Fisher JP. Biomechanical aspects of osteochondral regeneration: implications and strategies for three-dimensional bioprinting. *Tissue Engineering Part B: Reviews* 2022;28:766-788.

32. Barui S, Ghosh D, Laurencin CT. Osteochondral regenerative engineering: challenges, state-of-the-art and translational perspectives. *Regenerative Biomaterials* 2023;10:rbac109.

33. Ramzan F, Salim A, Khan I. Osteochondral tissue engineering dilemma: scaffolding trends in regenerative medicine. *Stem Cell Reviews and Reports* 2023;19:1615-1634.

34. Wu X, Chen K, Chai Q, Zhang X, Feng H, Feng C, Wang D, Li X, Zhang D. A 3D printed multilayer biomimetic scaffold with a gradient-oriented structure for articular cartilage repair. *Journal of Materials Chemistry B* 2025;13:7728-7743.

35. Kumar R, Sporn K, Prabhakar P, Paladugu P, Khanna A, Ngo A, Gowda C, Waisberg E, Jagadeesan R, Zaman N. Next-Generation Biomaterials for Load-Bearing Tissue Interfaces: Sensor-Integrated Scaffolds and Mechanoadaptive Constructs for Skeletal Regeneration. *Journal of Functional Biomaterials* 2025;16:232.

36. Peng Y, Zhuang Y, Liu Y, Le H, Li D, Zhang M, Liu K, Zhang Y, Zuo J, Ding J. Bioinspired gradient scaffolds for osteochondral tissue engineering. In. *Exploration*: Wiley Online Library; 2023, 20210043.

37. Beeren IA, Dijkstra PJ, Mota C, Camarero‐Espinosa S, Baker MB, Moroni L. Advances in Additive Manufactured Scaffolds Mimicking the Osteochondral Interface. *Advanced NanoBiomed Research* 2024;4:2400059.

38. Zhao Y, Ding X, Dong Y, Sun X, Wang L, Ma X, Zhu M, Xu B, Yang Q. Role of the Calcified Cartilage Layer of an Integrated Trilayered Silk Fibroin Scaffold Used to Regenerate Osteochondral Defects in Rabbit Knees. *ACS Biomaterials Science & Engineering* 2020;6:1208-1216.

39. Di Martino A, Salerno M, Galassi E, Grillini L, Dotti A, De Luca C, Filardo G. Osteochondral regeneration with a tri-layered biomimetic resorbable scaffold: In vivo study in a sheep model up to 12 months of follow-up. *Biomaterials* 2025;314:122821.

40. Zhong Y, Cao X, Huang M, Lei Y, Liu A-L. Biomimetic bone cartilage scaffolds based on trilayer methacrylated hydroxyapatite/GelMA composites for full-thickness osteochondral regeneration. *International Journal of Biological Macromolecules* 2025;298:139860.

41. Wu D, Zheng K, Yin W, Hu B, Yu M, Yu Q, Wei X, Deng J, Zhang C. Enhanced osteochondral regeneration with a 3D-Printed biomimetic scaffold featuring a calcified interfacial layer. *Bioactive Materials* 2024;36:317-329.

42. Zhang M, Meng N, Wang X, Chen W, Zhang Q. TRPV4 and PIEZO channels mediate the mechanosensing of chondrocytes to the biomechanical microenvironment. *Membranes* 2022;12:237.

43. Agarwal P, Lee H-p, Smeriglio P, Grandi F, Goodman S, Chaudhuri O, Bhutani N. A dysfunctional TRPV4–GSK3β pathway prevents osteoarthritic chondrocytes from sensing changes in extracellular matrix viscoelasticity. *Nature biomedical engineering* 2021;5:1472-1484.

44. Chen C, Yang F, Chen R, Yang C, Xiao H, Geng B, Xia Y. TRPV channels in osteoarthritis: a comprehensive review. *Biomolecules* 2024;14:292.

45. Sun K, Luo J, Guo J, Yao X, Jing X, Guo F. The PI3K/AKT/mTOR signaling pathway in osteoarthritis: a narrative review. *Osteoarthritis and cartilage* 2020;28:400-409.

46. Klampfleuthner FA, Lotz B, Renkawitz T, Richter W, Diederichs S. Stage-dependent activity and pro-chondrogenic function of PI3K/AKT during cartilage neogenesis from mesenchymal stromal cells. *Cells* 2022;11:2965.

47. Lin W, Kang H, Niu Y, Niu J, Fan C, Feng X, Wang F. Cartilage degeneration is associated with activation of the PI3K/AKT signaling pathway in a growing rat experimental model of developmental trochlear dysplasia. *Journal of Advanced Research* 2022;35:109-116.

48. Zhao R, Chen J, Li Y, Qian H, Zhu X, Maria GR, Ambrosio L, Yang X, Zhang X. Hierarchical micro-/nanostructured hydroxyapatite scaffolds promote osteoporotic bone regeneration via activation of hedgehog and HIF-1α signaling. *Bioactive Materials* 2026;60:640-660.

49. Elsaid KA, Zhang LX, Zhao T, Marks A, Jenkins D, Schmidt TA, Jay GD. Proteoglycan 4 (Lubricin) and regulation of xanthine oxidase in synovial macrophage as a mechanism of controlling synovitis. *Arthritis Research & Therapy* 2024;26:214.

50. Ignatyeva N, Gavrilov N, Timashev PS, Medvedeva EV. Prg4-Expressing Chondroprogenitor Cells in the Superficial Zone of Articular Cartilage. *International Journal of Molecular Sciences* 2024;25:5605.

51. Markway BD, Cho H, Zilberman-Rudenko J, Holden P, McAlinden A, Johnstone B. Hypoxia-inducible factor 3-alpha expression is associated with the stable chondrocyte phenotype. *Journal of Orthopaedic Research* 2015;33:1561-1570.

52. Lepage SI, Robson N, Gilmore H, Davis O, Hooper A, St. John S, Kamesan V, Gelis P, Carvajal D, Hurtig M. Beyond cartilage repair: the role of the osteochondral unit in joint health and disease. *Tissue Engineering Part B: Reviews* 2019;25:114-125.

53. Davis S, Roldo M, Blunn G, Tozzi G, Roncada T. Influence of the mechanical environment on the regeneration of osteochondral defects. *Frontiers in Bioengineering and Biotechnology* 2021;9:603408.
