# Supplementary material for: Three-dimensionally-printed biphasic PCL/β-TCP scaffold with spatially confined GelMA/CS hydrogel for coordinated osteochondral regeneration
Source: Regen Biomater. 2026 Jun 5;13:rbag111. doi: 10.1093/rb/rbag111 (PMC13303298; doi:10.1093/rb/rbag111)
Supplement: rbag111_Supplementary_Data [file rbag111_supplementary_data.zip › SI3.docx]

**3D-Printed Biphasic PCL/β-TCP Scaffold with Spatially Confined GelMA/CS Hydrogel for Coordinated Osteochondral Regeneration**

Feng Zhou^1, 2^, Xiaoyun Pan^3^, Qixiang Yin^2^, Xiao Yang^4,*^, Xiangdong Zhu^4^, Maria Grazia Raucci^5^, Luigi Ambrosio^5^, Xingdong Zhang^4^, Jingyi Mi^3,*^

^1^ Suzhou Medical College of Soochow University, Suzhou 215123, China;

^2^ Department of Emergency Surgery, Affiliated Hospital of Jiangsu University, Zhenjiang 212001, China;

^3^ Wuxi Ninth People's Hospital, Affiliated to Soochow University, Wuxi 214000, China;

^4^ National Engineering Research Center for Biomaterials, Sichuan University, Chengdu 610064, China

^5^ Institute of Polymers, Composites and Biomaterials, National Research Council, Naples 80072, Italy

*Corresponding Authors

Tel.: 86-28-85417654;

Fax: 86-28-85410246

E-mail address: xiaoyang114@scu.edu.cn (Xiao Yang) and mijingyi@suda.edu.cn (Jingyi Mi)


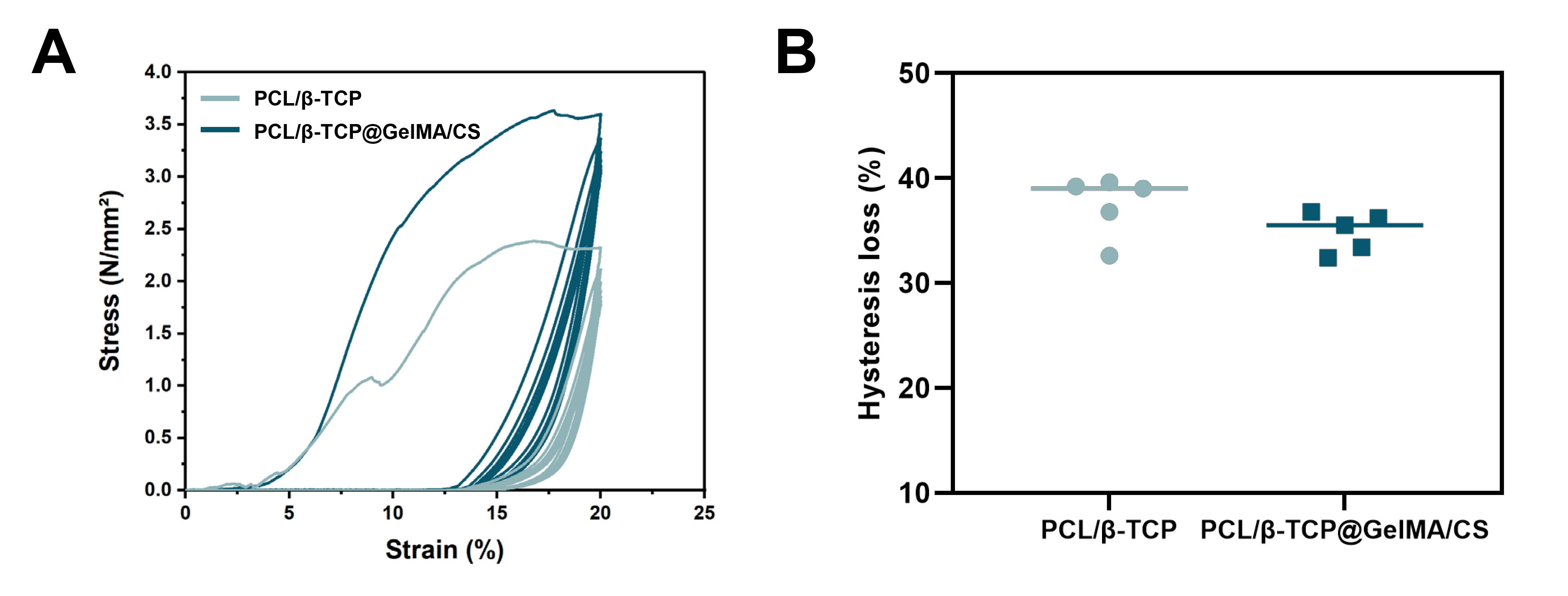


**Figure S1.** Cyclic compression behavior of representative scaffold constructs. (A) Representative cyclic compression stress–strain loops of the scaffold-only and hydrogel-integrated constructs. The first loading cycle was treated as a conditioning cycle because porous constructs showed initial seating and structural settling during loading. After conditioning, both constructs exhibited reproducible hysteresis loops under repeated compression. (B) Quantification of hysteresis loss (%) during cyclic loading, indicating viscoelastic energy dissipation in both constructs. These data provide supplementary construct-level mechanical information under repeated loading and indicate stable cyclic viscoelastic behavior in both scaffold-only and hydrogel-integrated constructs.

**
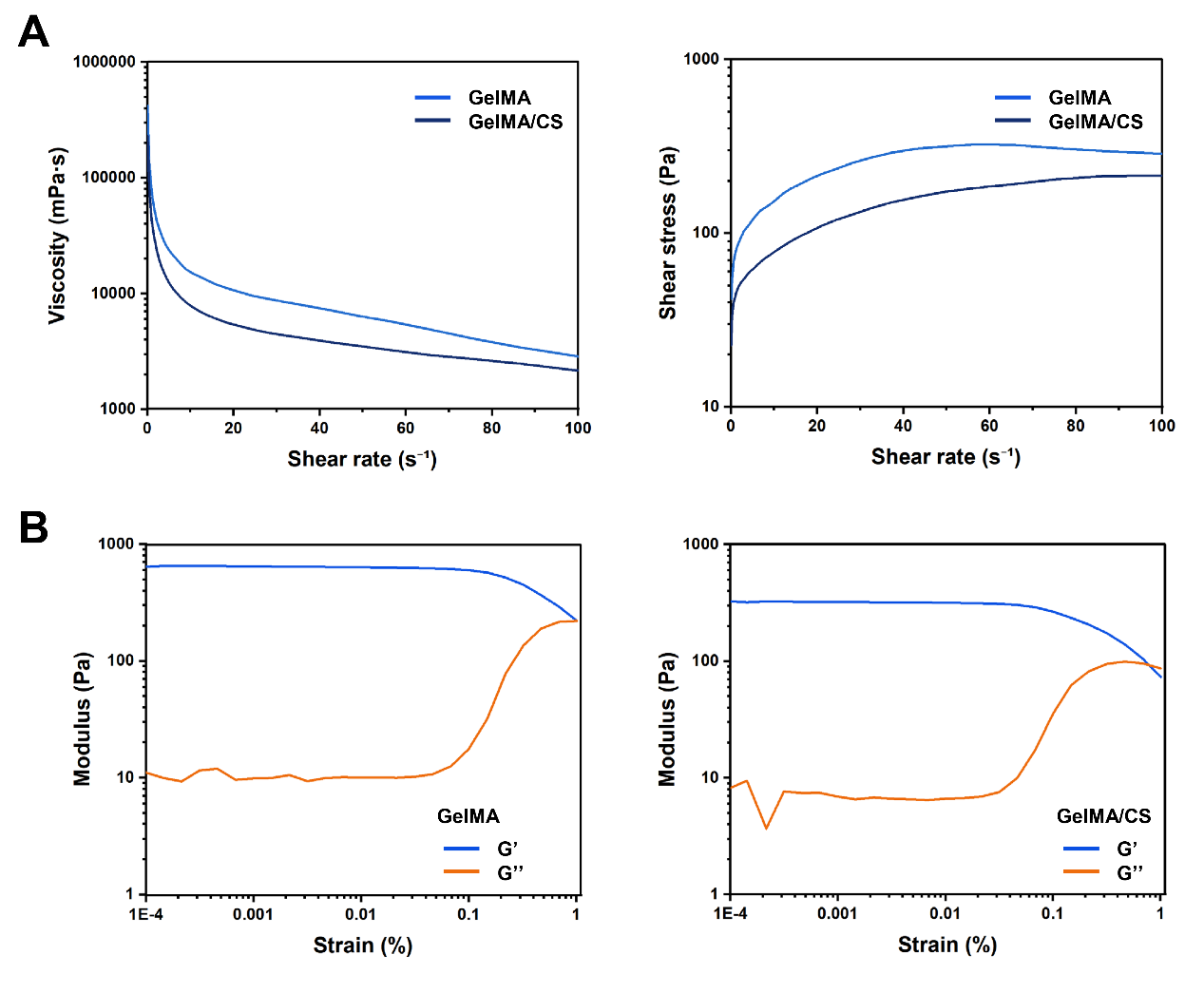
**

**Figure S2.** Rheological characterization of GelMA and GelMA/CS hydrogels. (A) Flow sweep analysis showing viscosity and shear stress as a function of shear rate. (B) Oscillatory strain sweep showing storage modulus (G′) and loss modulus (G″) as a function of strain.

**
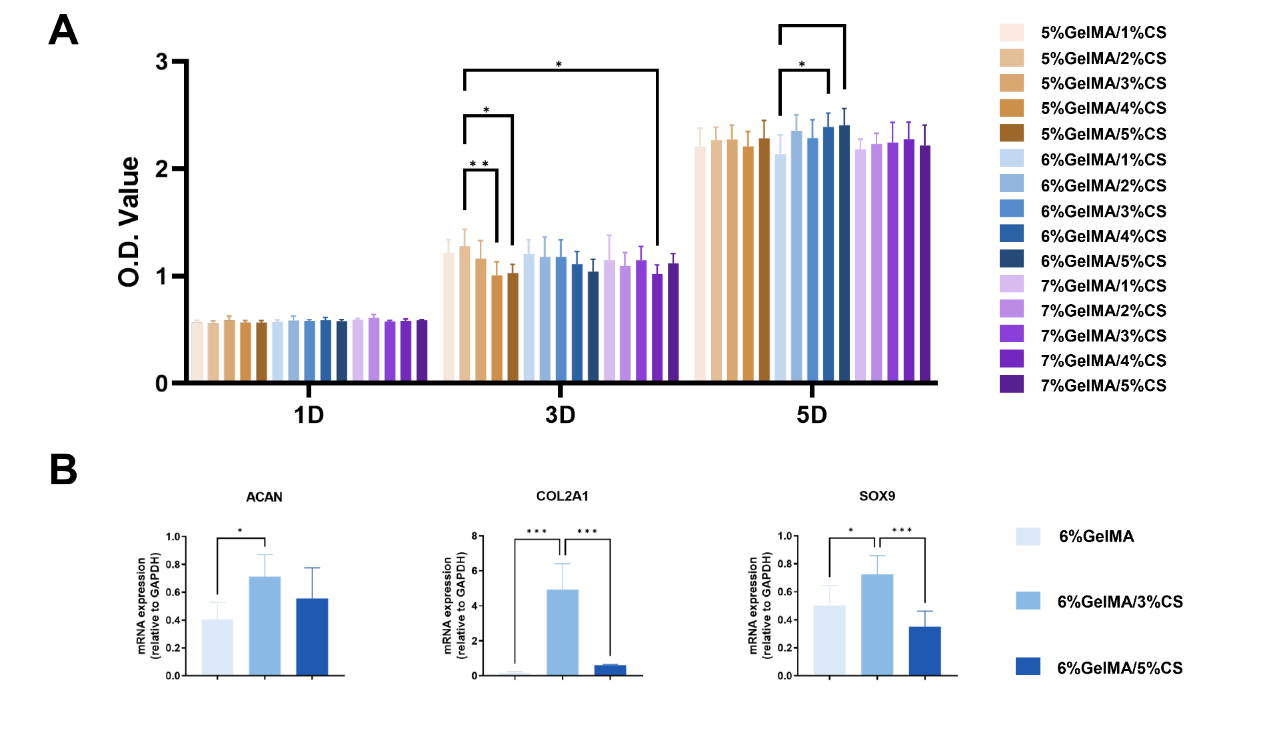
****Figure S3.** (A) CCK-8 assay of hUCMSCs cultured with hydrogel-conditioned medium prepared from GelMA/CS hydrogels containing 5%, 6%, or 7% GelMA (w/v) combined with different CS concentrations (1–5%, w/v). Metabolic activity was measured at days 1, 3, and 5. (B) qRT-PCR analysis of hUCMSCs treated with conditioned medium from 6% GelMA hydrogels supplemented with CS at 1%, 3%, or 5% (w/v). Chondrogenic gene expression (SOX9, COL2A1, and ACAN) was quantified.

**
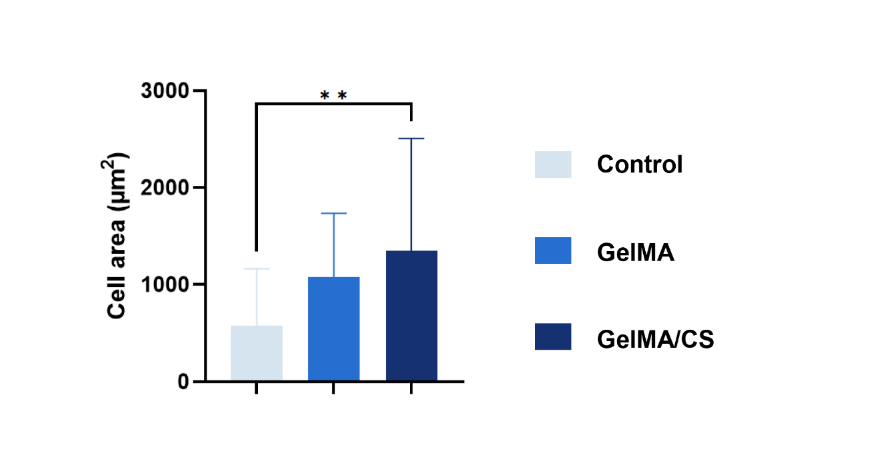
**

**Figure S4.** Quantification of single-cell spreading area of hACs on GelMA and GelMA/CS hydrogel surfaces. Cell area was measured in ImageJ based on F-actin outlines.

**
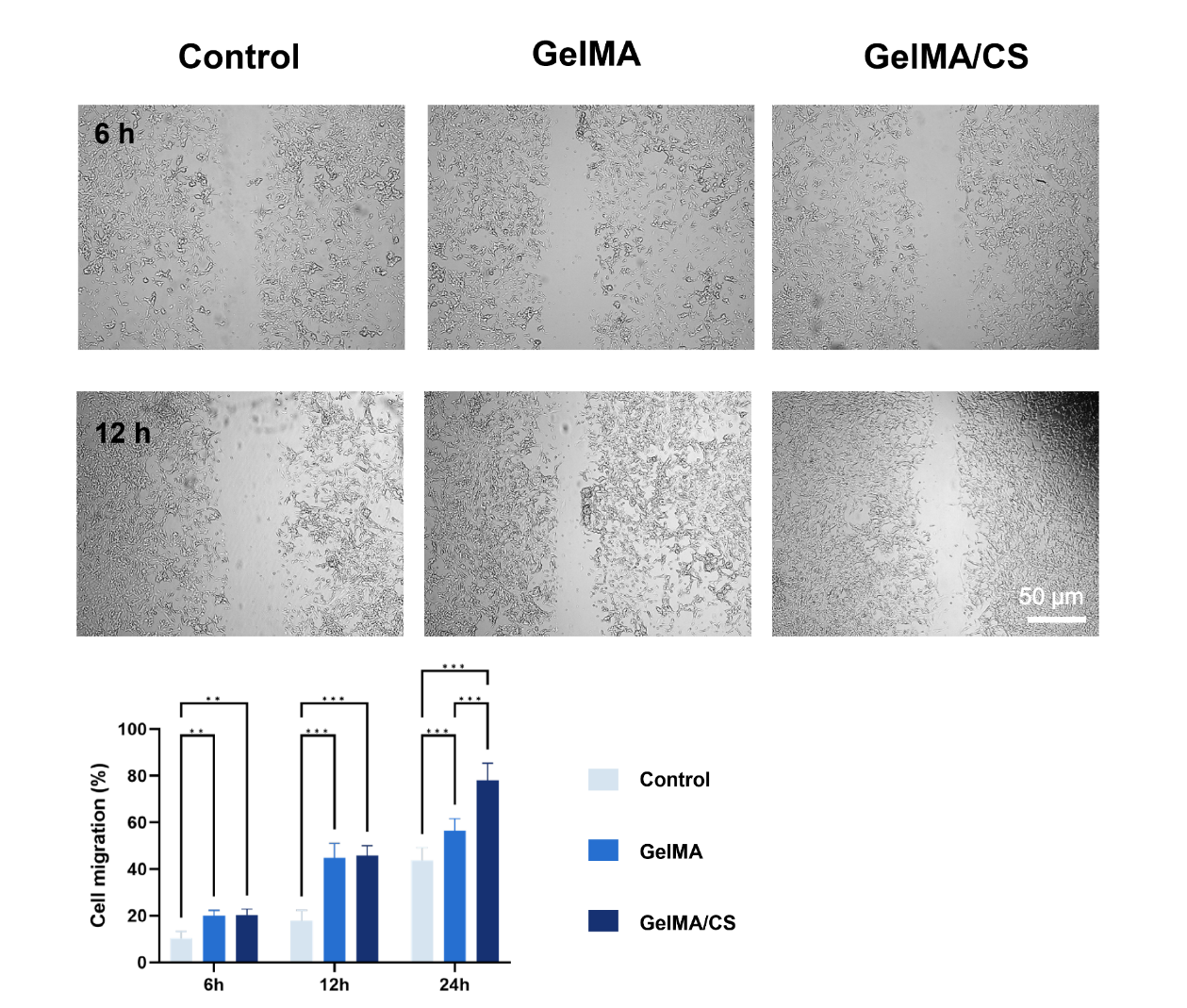
Figure S5.** Time-dependent wound closure of hAC monolayers treated with hydrogel extracts. Representative images from a scratch wound healing assay at 6 h and 12 h after scratching, showing hAC wound closure in response to hydrogel extracts from GelMA or GelMA/CS formulations. Hydrogel extracts were prepared by pre-incubating GelMA or GelMA/CS hydrogels in low-serum medium and then applied to scratched hAC monolayers. Both hydrogel extracts accelerated wound closure compared with the control, with greater closure observed in the GelMA/CS group. Wound closure (%) was quantified in ImageJ relative to 0 h.

**
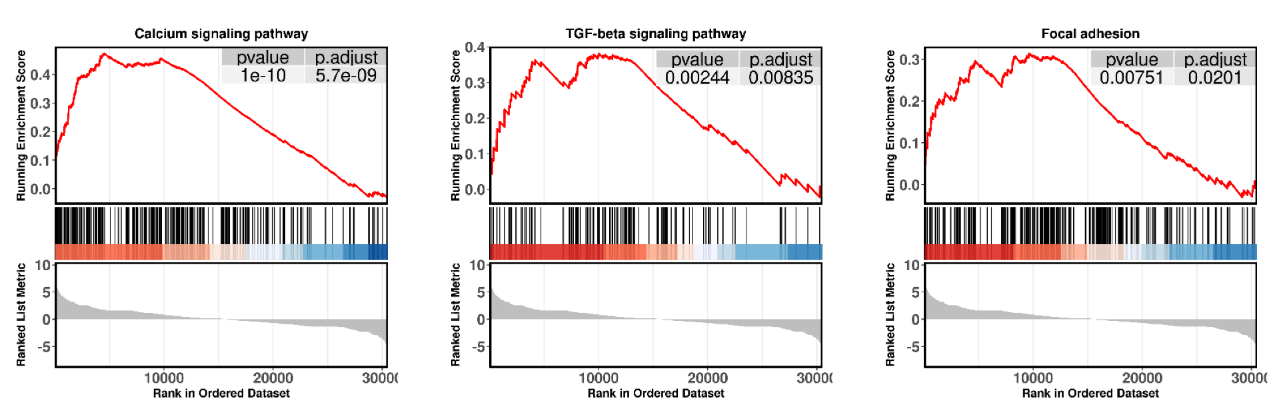
Figure S6.** Representative gene set enrichment analysis (GSEA) plots showing significant enrichment of pathways related to TGF-β signaling, focal adhesion, and calcium ion transmembrane transport in hUCMSCs cultured on PCL/β-TCP@GelMA/CS scaffolds relative to scaffold-free controls.

**
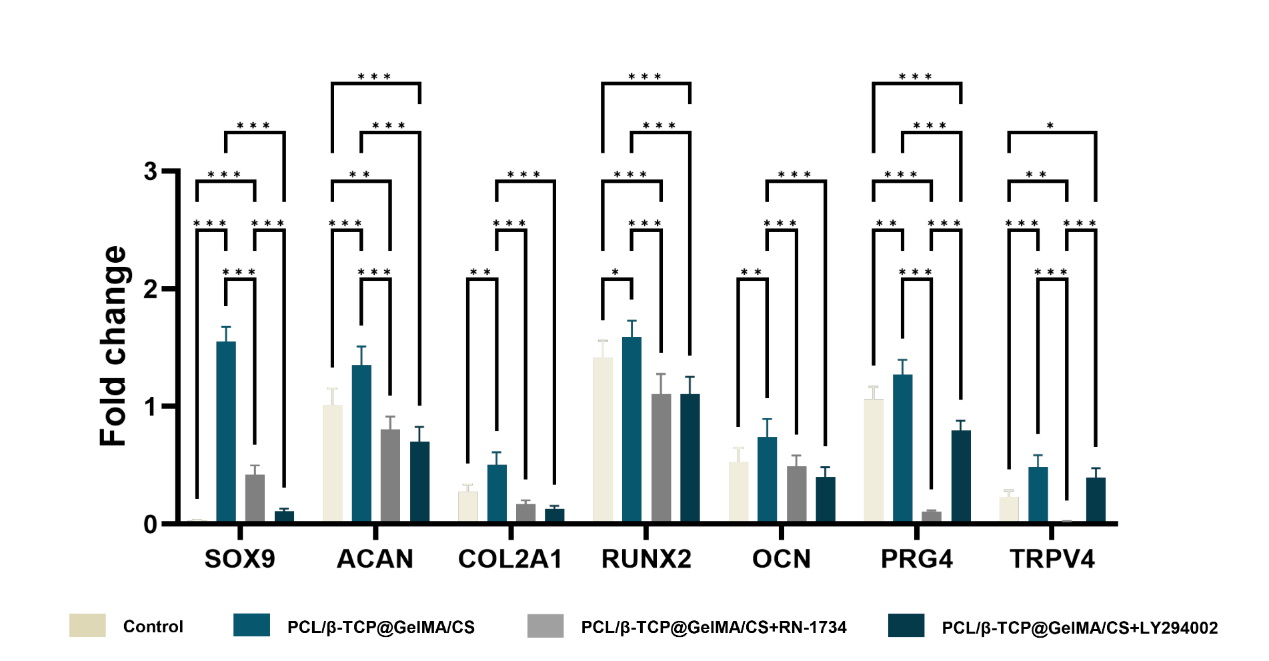
**

**Figure S7.** Semi-quantitative densitometric analysis of Western blot results for TRPV4, PRG4, SOX9, ACAN, COL2A1, RUNX2, and OCN. Protein expression levels were normalized to GAPDH.


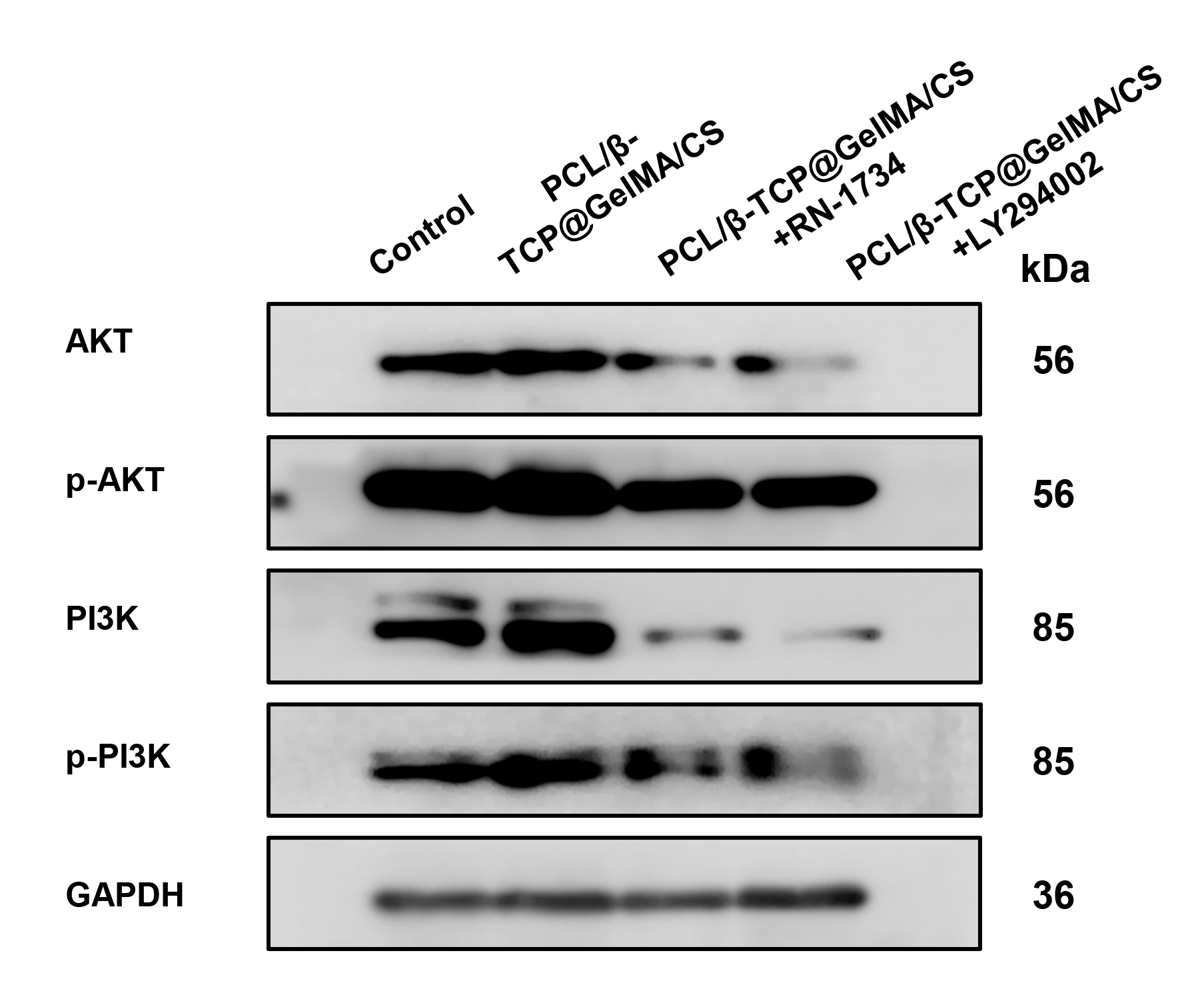


**Figure S8.** Western blot analysis of PI3K/AKT-related pathway activation under inhibitor treatment conditions. Representative blots of PI3K, p-PI3K, AKT, p-AKT, and GAPDH in the control, PCL/β-TCP@GelMA/CS, PCL/β-TCP@GelMA/CS + RN-1734, and PCL/β-TCP@GelMA/CS + LY294002 groups. These phosphorylation data provide supplementary support for the involvement of TRPV4-associated mechanosensing and PI3K/AKT-related signaling in the scaffold-induced cellular response.

**
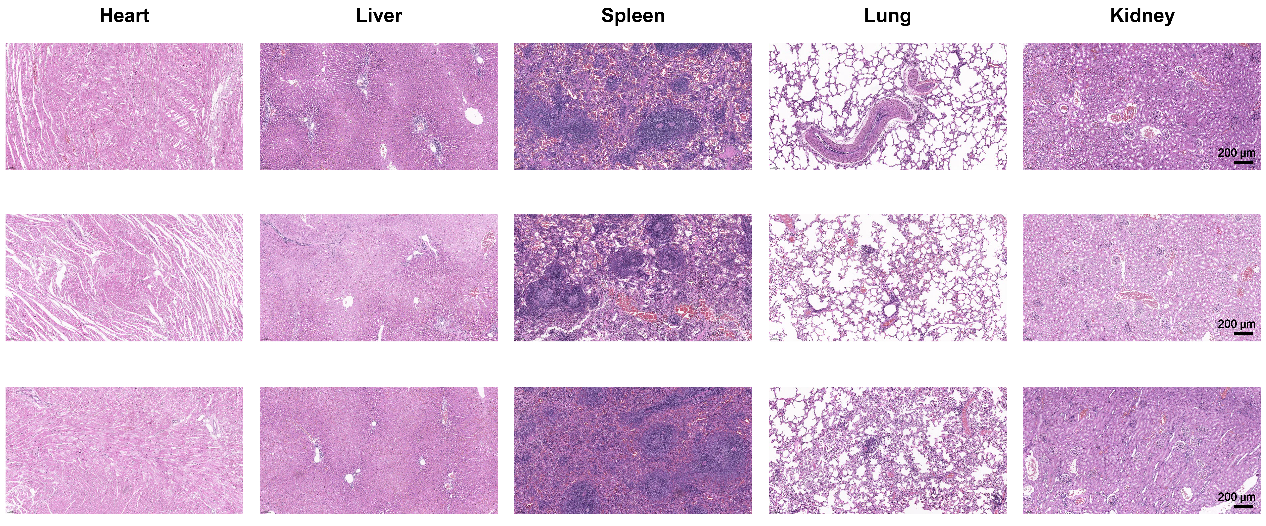
**

**Figure S9.** Preliminary in vivo biosafety evaluation. Representative H&E staining images of the heart, liver, spleen, lung, and kidney harvested at the experimental endpoint are shown. No obvious pathological abnormalities were observed. Because the in vivo study used a bilateral paired implantation design, these major-organ images are presented as a descriptive assessment of overall systemic biocompatibility rather than a strict material-specific comparison.

Table S1. Primer sequences for qRT-PCR analysis of genes.

| **Target** | **Forward primer** | **Reverse primer** |
| --- | --- | --- |
| SOX9  COL2  ACAN  COMP  COL10  COL1  BMP2  OCN  RUNX2  OPN  GLI1  TRPV4  PRG4  HIF3A  FGFR3  FGF18  β-ACTIN | GGCAAGCTCTGGAGACTTCTG  ACCAGGACCAAAGGGACAGA  GTGGTGATGATCTGGCACGA  GGCATCCAACTCAAGGCTGT  ACTCCCAGCACGCAGAATC  GGAATGAAGGGACACAGAGGTT  GGAACGGACATTCGGTCCTT  CCACCGAGACACCATGAGAG  CGCCTCACAAACAACCACAG  GCAGCTTTACAACAAATACCCA  GCCTGTTCTAATGGTGCCAAC  TCCCGCAAGTTCAAGGACTG  GTTTCATCTCAAGAGCTTTCCTGT  CGCAGCTGGAGCTCATT  AGGAGCTCTTCAAGCTGCTG  GGGACAAGTATGCCCAGCTC  CACGAAACTACCTTCAACTCC | CGCCTTGAAGATGGCGTTG  ACCTTTGTCACCACGATCCC  GTTTGTAGGTGGTGGCTGTG  CGCATGGTTGTGTCCAAGAC  TGCCTGTGGGCATTTGGTAT  GTAGCACCATCATTTCCACGA  CACCATGGTCGACCTTTAGGA  TTGGACACAAAGGCTGCAC  ACTGCTTGCAGCCTTAAATGAC  ACTTACTTGGAAGGGTCTGTGG  CCACACTCGCACACATAGG  TCTCGTGGCGGTTCTCAATC  TGTGGGATTATGCACTTCTGC  AGAGCAGTTCAGCACCTTCC  AGGTCCAGGTACTCGTCGGT  GGCCGTGTAGTTGTTCTCCA  CATACTCCTGCTTGCTGATC |
