# Supplementary material for: Three-dimensionally-printed biphasic PCL/β-TCP scaffold with spatially confined GelMA/CS hydrogel for coordinated osteochondral regeneration
Source: Regen Biomater. 2026 Jun 5;13:rbag111. doi: 10.1093/rb/rbag111 (PMC13303298; doi:10.1093/rb/rbag111)
Supplement: rbag111_Supplementary_Data [file rbag111_supplementary_data.zip › Response to reviewers2.docx]

**Response to reviewers**

**Reviewer: 1**

Comments to the Author
The authors have made substantial revisions in response to the previous comments. The revised manuscript is generally improved, particularly in clarifying the hAC and hUCMSC experimental timelines, correcting the RNA-seq comparison groups, moderating the TRPV4/PI3K-AKT mechanistic interpretation, and framing the in vivo findings as early-stage osteochondral repair rather than definitive long-term regeneration.

Before acceptance, I suggest a final round of minor textual and consistency revisions:

1. Please carefully check that the corrected hAC and hUCMSC assay descriptions, time points, and figure legends are fully consistent throughout the Methods, Results, figures, and supplementary materials.

**Response:** We thank the reviewer for this careful and helpful suggestion. We have rechecked the revised manuscript once again to ensure consistency of the hAC and hUCMSC experimental descriptions, assay time points, figure legends, and supplementary figure descriptions throughout the Methods, Results, figures, and supplementary materials. In response to this comment, we further revised the title and description of Section 2.4 to clarify that the hAC experiments were performed on GelMA and GelMA/CS hydrogel samples rather than on hydrogel-modified scaffolds. The control condition for the hAC experiments was also explicitly defined as hACs cultured on tissue-culture plastic under the same seeding density and culture conditions. In addition, because F-actin was visualized as green fluorescence, we corrected the staining reagent from rhodamine-conjugated phalloidin to FITC-conjugated phalloidin in the Methods. The Figure 3 legend was also revised accordingly to clearly state that hACs were cultured on tissue-culture plastic control, GelMA, or GelMA/CS hydrogel surfaces.

Specifically, for the hAC experiments, cell proliferation is now consistently described as being assessed by CCK-8 at days 1, 4, and 7; cytoskeletal organization was assessed at 48 h; and sGAG production was measured at days 14 and 21 and normalized to total DNA content. For the hUCMSC experiments, proliferation is consistently described as being assessed by CCK-8 at days 1, 3, and 5, and sGAG production was measured at day 7 and normalized to total DNA content. We also rechecked the preliminary hydrogel formulation screening shown in Supplementary Figure S3 and confirmed that this experiment was performed using hUCMSCs cultured with hydrogel-conditioned medium, whereas the subsequent direct hydrogel-response experiments in Figure 3 were performed using hACs. The relevant text, figure legends, and supplementary descriptions have been carefully proofread and aligned accordingly. Representative revisions include:

“2.4 In vitro chondrocyte culture on GelMA and GelMA/CS hydrogels”

“For the control group, hACs were cultured on tissue-culture plastic under the same seeding density and culture conditions.”

“Cell proliferation was assessed using the Cell Counting Kit-8 (CCK-8) assay at days 1, 4, and 7.”

“Cytoskeletal organization was assessed at 48 h after seeding.”

“F-actin was stained with FITC-conjugated phalloidin (1:200 dilution; Thermo Fisher Scientific) for 60 min at room temperature in the dark, and nuclei were counterstained with DAPI (1 μg mL⁻¹) for 5 min.”

“Cell proliferation was assessed using the Cell Counting Kit-8 (CCK-8; Dojindo) assay at days 1, 3, and 5.”

“At day 7, sGAG levels in culture supernatants were quantified…”

“Confocal immunofluorescence images of hACs cultured on tissue-culture plastic control, GelMA, or GelMA/CS hydrogel surfaces…”

2. Please ensure that the RNA-seq results are consistently described as a comparison between the no-scaffold control and the PCL/β-TCP@GelMA/CS group. The manuscript should avoid wording that implies the RNA-seq data specifically isolate the effect of GelMA/CS relative to the uncoated scaffold.
**Response:** We thank the reviewer for this important and constructive suggestion. We have carefully rechecked the manuscript, figures, and supplementary materials to ensure that the RNA-seq results are consistently described as a comparison between the no-scaffold control group and the PCL/β-TCP@GelMA/CS group. We agree that the RNA-seq analysis should not be interpreted as isolating the specific contribution of GelMA/CS relative to the uncoated scaffold.

Accordingly, we revised the wording in the Introduction, Methods, Results, and Figure 5 legend to clarify that the transcriptomic data reflect scaffold-associated cellular responses to the complete PCL/β-TCP@GelMA/CS construct relative to the no-scaffold control. Any wording that could imply a direct RNA-seq comparison between PCL/β-TCP@GelMA/CS and the uncoated PCL/β-TCP scaffold, or that could suggest isolation of the GelMA/CS-specific effect, has been avoided.

Representative revisions include:

“RNA sequencing was conducted to explore scaffold-associated transcriptomic programs related to osteogenic and chondrogenic responses.”

“To explore scaffold-associated transcriptomic changes in hUCMSCs, RNA sequencing (RNA-seq) was performed after 14 days of osteochondral induction. Two experimental groups were analyzed: (i) control (no scaffold) and (ii) PCL/β-TCP@GelMA/CS (biphasic scaffold with GelMA/CS hydrogel integrated in the cartilage phase).”

“Transcriptomic comparison was performed between the control group (no scaffold) and the PCL/β-TCP@GelMA/CS group after 14 days of osteochondral induction.”

“Differential expression analysis identified 11,836 significantly altered genes … in the PCL/β-TCP@GelMA/CS group relative to the control group, indicating a robust scaffold-associated transcriptomic shift.”

3. Please maintain cautious mechanistic wording throughout the manuscript. The current data support the involvement of TRPV4-associated mechanosensing and PI3K/AKT-related signaling, but should not be described as proving a definitive TRPV4–PI3K/AKT axis, direct crosstalk, or a strict upstream–downstream pathway.
**Response:** We thank the reviewer for emphasizing this important point. We fully agree that the current data support the involvement of TRPV4-associated mechanosensing and PI3K/AKT-related signaling, but do not establish a definitive TRPV4–PI3K/AKT axis, direct crosstalk, or a strict upstream–downstream relationship. We have therefore carefully reviewed the Abstract, Methods, Results, Figure 5 legend, Discussion, and Conclusion, and further revised the mechanistic wording to ensure a cautious and evidence-based interpretation.

Specifically, we revised the Results subsection title from a stronger validation-oriented wording to:

“3.5 Transcriptomic profiling and functional assessment of TRPV4- and PI3K/AKT-related signaling”

We also revised the Figure 5 legend to avoid implying definitive pathway validation:

“Figure 5. Transcriptomic profiling and inhibitor-based assessment of TRPV4- and PI3K/AKT-related signaling in hUCMSCs.”

“Inhibitor studies using RN-1734 (TRPV4) and LY294002 (PI3K) are consistent with the involvement of TRPV4-associated mechanosensing and PI3K/AKT-related signaling.”

In the Results section, we now describe the inhibitor and phosphorylation findings as associative evidence rather than proof of a fixed signaling cascade:

“These inhibitor- and phosphorylation-associated changes support the involvement of TRPV4-associated mechanosensing and PI3K/AKT-related signaling in the cellular response to the GelMA/CS-functionalized scaffold.”

We further revised the Discussion to explicitly acknowledge the limitations of the pharmacological inhibition experiments and to avoid claiming a strict hierarchical pathway:

“Within the limits of the present pharmacological inhibition setup, our data support the involvement of both TRPV4-associated mechanosensing and PI3K/AKT-related signaling in the response to the functionalized scaffold, without resolving their strict hierarchical relationship or relative dominance.”

In addition, we removed wording that could imply a definitive “TRPV4–PI3K/AKT-mediated mechanotransduction axis” and replaced it with more cautious wording:

“Together with transcriptomic and inhibitor-based data suggesting the involvement of TRPV4-associated mechanosensing and PI3K/AKT-related signaling, this modular platform offers a scalable and translationally relevant strategy for osteochondral defect repair.”

4. Please ensure that claims regarding the approximately 0.5 mm transition zone remain appropriately limited. The current evidence supports this as a structural design feature, but does not directly demonstrate reduced interfacial stress concentration or improved interfacial mechanical strength.
**Response:** We thank the reviewer for highlighting this important point. We fully agree that the approximately 0.5 mm hydrogel-free transition zone should be described as a structural design feature, and that the current data do not directly demonstrate reduced interfacial stress concentration or improved interfacial mechanical strength.

In response to this comment, we carefully reviewed the Abstract, Methods, Results, Figure 2 legend, Discussion, and Conclusion, and revised the relevant wording to keep the interpretation appropriately limited. The transition zone is now consistently described as a hydrogel-free PCL structural transition region generated by spatially confined top-down hydrogel infiltration, rather than as direct evidence of stress reduction or enhanced interfacial mechanical strength.

Specifically, in the Methods and Results, we describe the approximately 0.5 mm region as follows:

- “A ~0.5 mm uninfiltrated PCL transitional zone was deliberately retained between the hydrogel-filled cartilage region and the underlying bone-mimetic phase, forming a structural transition region between the chondral hydrogel compartment and the subchondral scaffold.”
- “An ~0.5 mm uninfiltrated PCL transitional zone was consistently preserved between the hydrogel-filled region and the underlying bone-mimetic phase, forming a defined structural transition region between the chondral hydrogel compartment and the subchondral scaffold.”

We also revised the Discussion to avoid implying direct mechanical validation of this transition zone. The revised wording now states:

- “This configuration was designed to spatially confine the hydrogel within the chondral compartment while maintaining a hydrogel-free PCL region between the hydrogel-filled and β-TCP-containing phases.”

In addition, we ensured that the mechanical data are described only at the construct level. The cyclic compression and rheological analyses are now presented as supporting construct-level behavior and hydrogel processability, without claiming direct measurement of interfacial shear strength, reduced stress concentration, or improved interfacial mechanical strength.

These revisions clarify that the approximately 0.5 mm hydrogel-free region represents a deliberate structural design feature for spatial hydrogel confinement within the biphasic construct. Any functional role related to interfacial mechanics is now presented cautiously as a design rationale rather than as a directly demonstrated mechanical outcome.

5. Please further proofread the manuscript for minor typographical, formatting, abbreviation, and terminology inconsistencies, especially across figure legends and supplementary figures.
Overall, the manuscript has been substantially improved, and the remaining issues appear to be minor revisions related mainly to textual precision and consistency.

**Response:** We thank the reviewer for this helpful comment and for recognizing the improvement of the revised manuscript. We have carefully proofread the entire manuscript and supplementary materials once again, with particular attention to typographical errors, formatting, abbreviations, terminology, figure legends, and supplementary figure descriptions.

During this final proofreading, we further standardized the terminology for hACs and hUCMSCs, clarified the distinction between hydrogel-only experiments and scaffold-based experiments, and aligned the descriptions of assay time points across the Methods, Results, figure legends, and supplementary materials. We also corrected the F-actin staining description to match the fluorescence channel used in the images, revised Figure 3 to clearly define the tissue-culture plastic control, and ensured consistent use of “sGAG normalized to total DNA content” where applicable.

In addition, we rechecked the terminology related to RNA-seq and mechanistic interpretation. The RNA-seq comparison is now consistently described as no-scaffold control versus PCL/β-TCP@GelMA/CS, and the mechanistic wording has been revised to use cautious terms such as “TRPV4-associated mechanosensing,” “PI3K/AKT-related signaling,” “inhibitor-based assessment,” and “are consistent with the involvement of,” rather than implying a definitive signaling axis or direct upstream–downstream pathway.

We also reviewed the descriptions of the approximately 0.5 mm hydrogel-free transition region and ensured that it is consistently presented as a structural design feature for spatial hydrogel confinement, without overstating its mechanical role. Figure legends and supplementary figure descriptions were further checked for consistent formatting of abbreviations, group names, time points, and statistical annotations.

Representative corrections and consistency revisions include:

- Standardizing hAC and hUCMSC experimental descriptions and time points.
- Clarifying that Section 2.4 describes hAC culture on GelMA and GelMA/CS hydrogels, not hydrogel-modified scaffolds.
- Defining the hAC control as tissue-culture plastic.
- Correcting F-actin staining to FITC-conjugated phalloidin.
- Standardizing sGAG/DNA normalization descriptions.
- Ensuring consistent use of PCL/β-TCP, PCL/β-TCP@GelMA, and PCL/β-TCP@GelMA/CS across the manuscript.
- Revising Figure 5 terminology from stronger validation wording to inhibitor-based assessment.
- Maintaining cautious wording for TRPV4-associated mechanosensing and PI3K/AKT-related signaling.
- Limiting the description of the ~0.5 mm transition zone to a hydrogel-free structural transition region.
